# Supplementary material for: High-spin state dynamics and quintet-mediated emission in intramolecular singlet fission
Source: Nat Commun. 2026 Jan 20;17:777. doi: 10.1038/s41467-025-67383-3 (PMC12823601; doi:10.1038/s41467-025-67383-3)
Supplement: Supplementary file 1 — Supplementary Information [file 41467_2025_67383_MOESM1_ESM.pdf]

SUPPORTING INFORMATION

for

High-Spin State Dynamics and Quintet-Mediated Emission in Intramolecular Singlet Fission

Jeannine Grüne[1,2,6,*], Steph Montanaro[3], Thomas W. Bradbury[4], Ashish Sharma[1], Simon Dowland[1], Alexander J. Gillett[1], Sebastian Gorgon[1], Oliver Millington[3], William K. Myers[2], Jan Behrends[5], Jenny Clark[4], Akshay Rao[1], Hugo Bronstein[1,3], Neil C. Greenham[1,*]

[1]*Cavendish Laboratory, University of Cambridge, Cambridge CB3 0HE, U.K.*

[2]*Centre for Advanced Electron Spin Resonance, University of Oxford, Oxford OX1 3QR, U.K.*

[3]*Department of Chemistry, University of Cambridge, Cambridge CB2 1EW, U.K.*

[4]*Department of Physics and Astronomy, University of Sheffield, Sheffield S3 7RH, U.K.*

[5]*Fachbereich Physik, Freie Universität Berlin, 14195 Berlin, Germany.*

[6]*Present address: Department of Materials, University of Oxford, Oxford OX1 3PH, U.K.*

*Corresponding authors. Email addresses: jeannine.grune@materials.ox.ac.uk (Jeannine Grüne), ncg11@cam.ac.uk (Neil C. Greenham)

Table of Contents

Supplementary Note 1. Supplementary experimental results 2
Supplementary Note 2. EasySpin Simulation Parameter for trEPR and ODMR 8
Supplementary Note 3. Complementarity of TA, trEPR, trPL and ODMR techniques 10
Supplementary Note 4. Optical characterization of Me-(DPH)$_3$ 12
Supplementary Note 5. Mechanisms of Singlet-Quintet Mixing in Intramolecular Singlet Fission .. 14
Supplementary Note 6. Distinguish triplet states generated by singlet fission or ISC 15
Supplementary Note 7. Influence of Triplet-Triplet-Annihilation (TTA) 17
Supplementary Note 8. Magnetic field Effect 18
Supplementary Note 9. Spin States in DPH Systems 19
Supplementary Note 10. Kinetic Model 20
Supplementary Note 11. Comparison to other Singlet Fission Systems 22
Supplementary Note 12. Synthetic Details 23

Supplementary Note 1. Supplementary experimental results

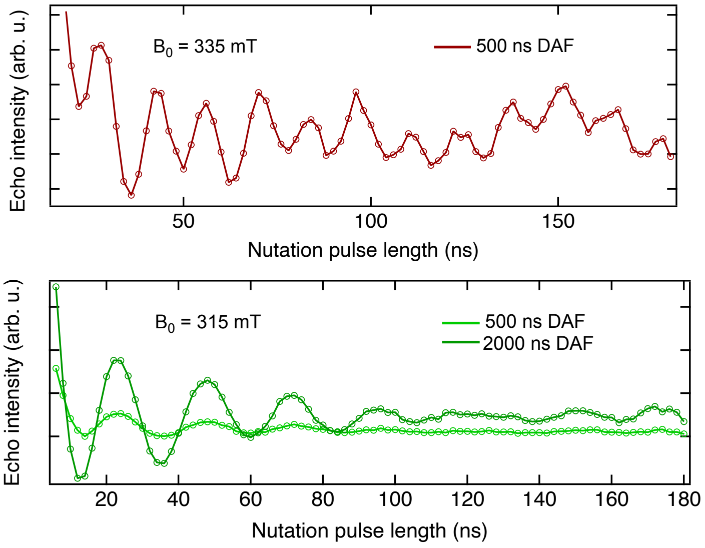

Supplementary Fig. 1. Transient nutation experiments on (DPH)$_3$. Nutation experiments were performed on quintet transition (B = 335 mT) and triplet transition (B = 315 mT). The ratio of nutation frequency $\omega_Q/\omega_T = 1.68$ exhibit the expected ratio of quintet to triplet formation.[1] The triplet signal increases over time, visible by a bigger response for 2μs delay after flash (DAF).

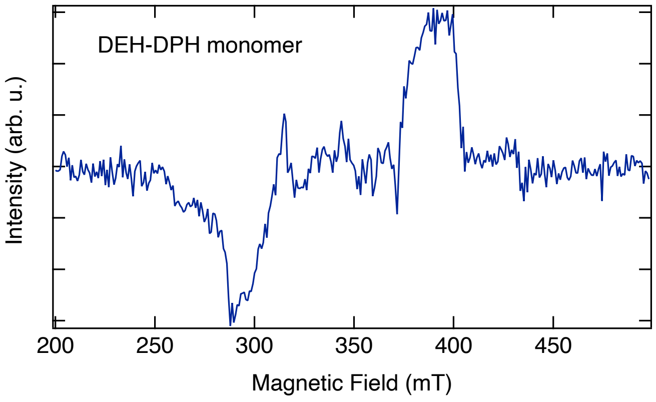

Supplementary Fig. 2. TrEPR of DEH-DPH. TrEPR of DEH-DPH diluted in toluene, showing the same triplet signal and zero field splitting as (DPH)$_2$ and ISC triplet of (DPH)$_3$. As no SF can occur in the monomer itself, it can only be attributed to ISC, fitting with the occupation of ZF states.

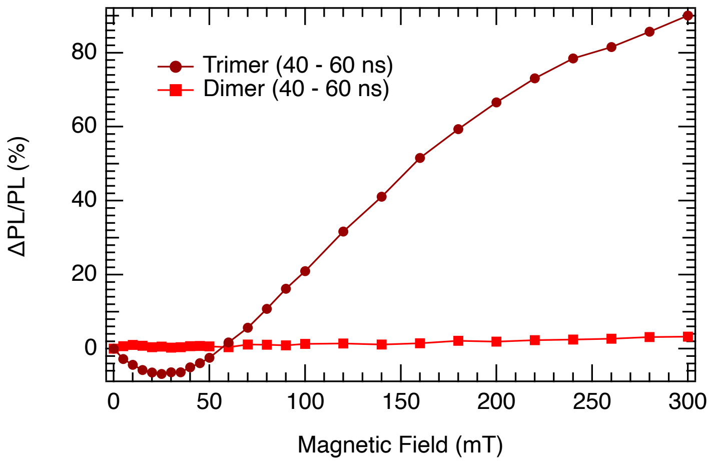

Supplementary Fig. 3. Time dependent MFE of dimer (DPH)$_2$ and trimer Me-(DPH)$_3$. Whilst the trimer shows a pronounced MFE at 40-60 ns after laser excitation, the dimer shows no MFE response. This confirms that the dimer does not enter persistently the weakly-coupled triplet pair regime.

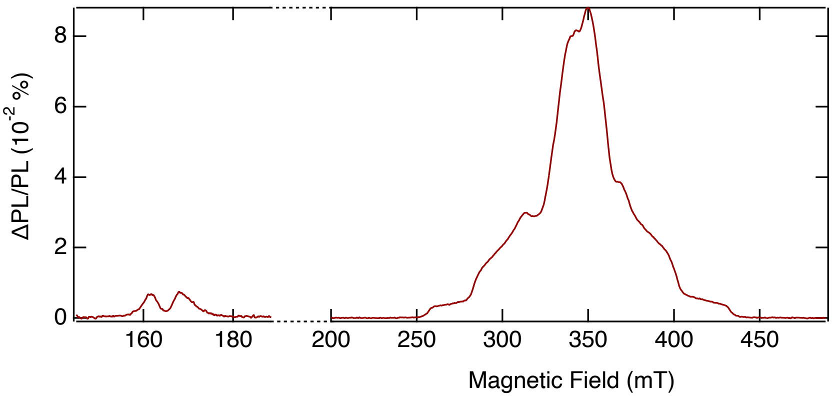

Supplementary Fig. 4. HF and FF ODMR of Me(DPH)$_3$. HF signal (cantered around B = 165 mT) and FF signal (cantered around B = 350 mT) of Me-(DPH)$_3$. The signals in the HF region of ODMR appear at the same positions as in the HF signal of trEPR (see Fig. 1b, main text).

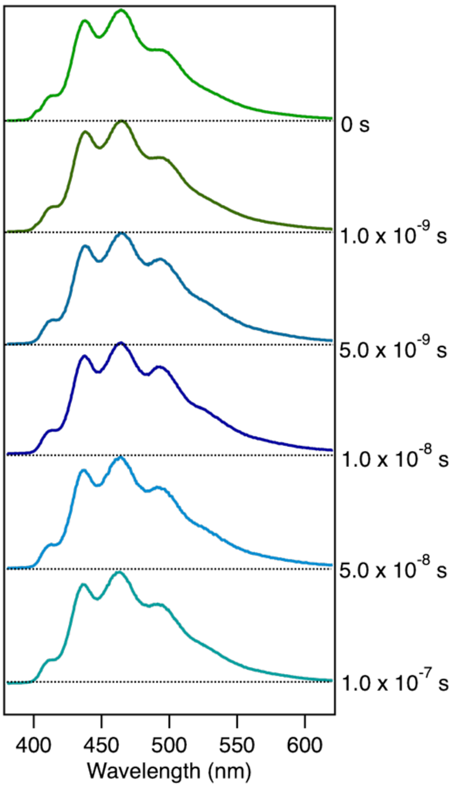

Supplementary Fig. 5. Time-dependent PL spectra. Time-dependent PL spectra with times after laser excitation displayed on the right. PL does not change over time, confirming the emission from the same S_1 state by recycled triplet pairs and high-spin states.

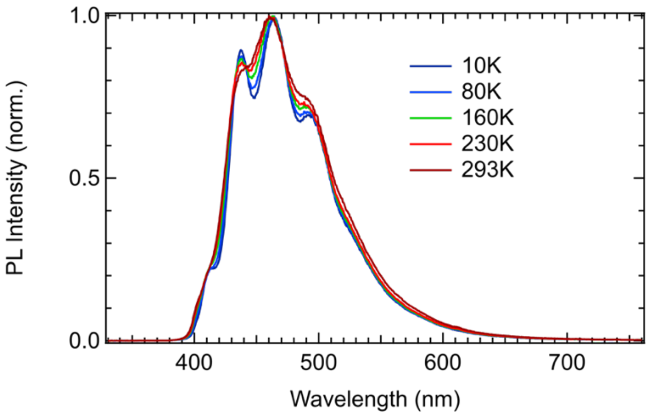

Supplementary Fig. 6. Temperature dependent PL spectra. Temperature dependent PL spectra show slight changes with temperatures, with a small reduction vibronic structure and S0-S2 intensity at higher temperatures.

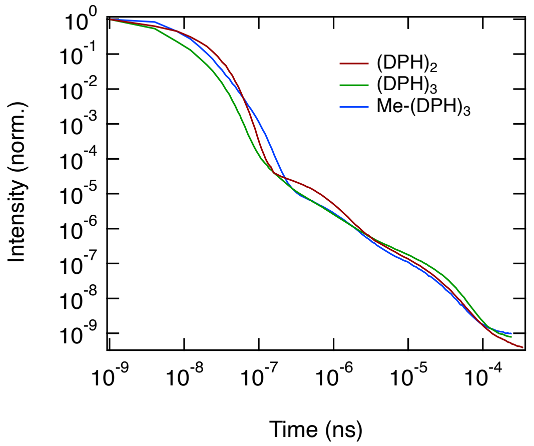

Supplementary Fig. 7. Transient PL for all three oligomers (DPH)$_2$, Me-(DPH)$_3$ and (DPH)$_3$. TrPL decay of all three oligomers are comparable up to several hundred μs. Whilst all show equivalent generation of quintet states, the dimer does not persistently enter the weakly-coupled regime. The similarity of trPL in all oligomers at ambient conditions hints that quintet states dominate the emission as high-spin reservoir at room temperature.

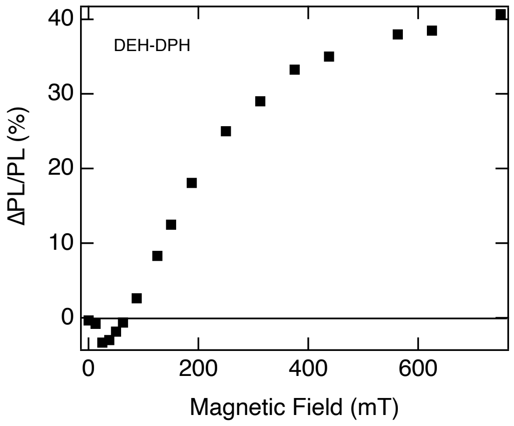

Supplementary Fig. 8. Magnetic field effect (MFE) of DEH-DPH film at room temperature. MFE of DEH-DPH films measured at room-temperature upon photoexciting under continuous illumination of 400 nm. The shape and zero-field crossing is comparable to Me-(DPH)$_3$.

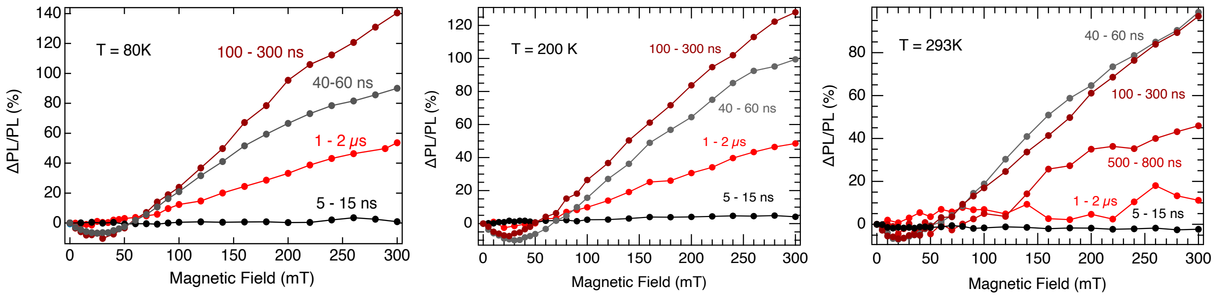

Supplementary Fig. 9. Time dependent MFE of Me-(DPH)$_3$ at 80K, 200K and 293K. The overall MFE effect reduces slightly and the timescales changes, as visible from integrated PL. At 293K, the magnetic field effect at 1-2μs is almost not measurable due to the faster decaying PL.

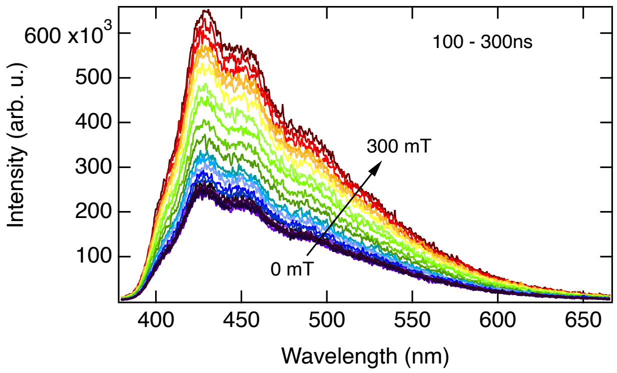

Supplementary Fig. 10. PL spectra of Me-(DPH)$_3$ at 80K for time window 100 – 300ns after laser pulse. PL spectra from 0mT to 300mT for 100 – 300ns window after laser pulse. Spectra measured with increasing and decreasing magnetic field. Shown here exemplary for 100 – 300ns and increasing field, analogue to other time windows.

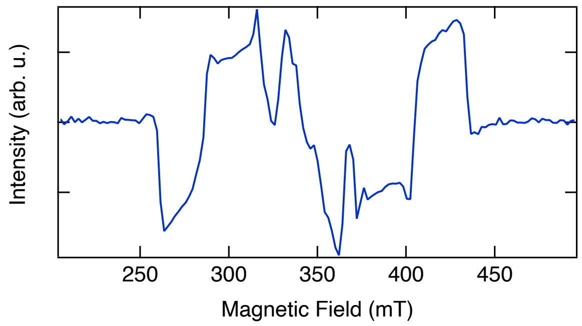

Supplementary Fig. 11. TrEPR of (DPH)$_3$ at 10K. TrEPR at 10K confirms that (DPH)$_3$ forms both quintet and triplet states via singlet fission at low temperature. In contrast to Me-(DPH)$_3$, where ISC emerges only at lower temperatures, ISC in (DPH)$_3$ is already present at higher temperatures and remains detectable at 10 K.

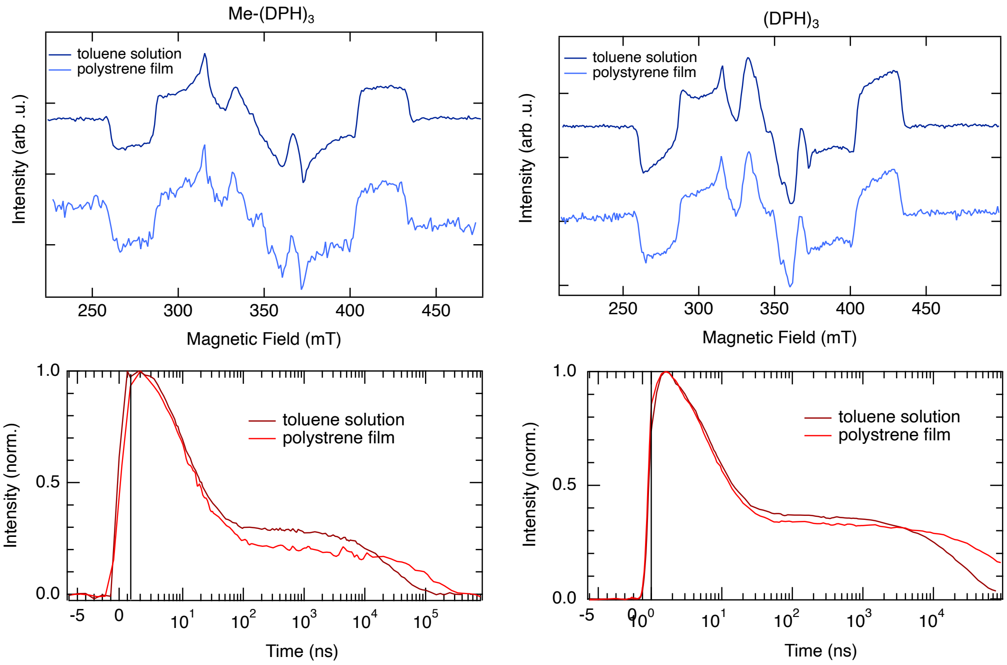

Supplementary Fig. 12. Comparison of transient EPR (top) and transient absorption (bottom) measurements of Me-(DPH)$_3$ and (DPH)$_3$ in frozen toluene solution and polystyrene film. Both sample formats show qualitatively identical spectral features and kinetic behaviour, confirming that the underlying spin dynamics are preserved across environments. Minor differences in signal-to-noise (EPR) and plateau amplitude (TA) arise from differences in laser power and sample optical density, respectively.

Supplementary Note 2. EasySpin Simulation Parameter for trEPR and ODMR

Transient Electron Paramagnetic Resonance

Supplementary Table 1. Simulation parameter for trEPR spectral simulations using the MATLAB toolbox EasySpin. Parameters given are the ZFS parameters *D* and *E*, relative populations, and the spectral linewidth with Gaussian and Lorentzian contributions [Gaussian Lorentzian]. The populations given for quintet and triplet by SF are the high-field populations, whilst ISC-generated triplet shows zero-field populations.

	**Quintet**		
**Material**	**|D|, |E| [MHz]**	**p_{-2},p_{-1},p_0,p_1, p_2**	**LW**
$(DPH)_2$	890, 0		5.0, 0
Me-$(DPH)_3$	750, 80	0, 0.1, 1.0, 0.1, 0	3.0, 0
$(DPH)_3$	780, 90	0, 0.1, 1.0, 0.1, 0	3.0, 0

	**Triplet by SF**			**Triplet by ISC**		
**Material**	**|D|, |E| [MHz]**	**p_{-1},p_0,p_1**	**LW**	**|D|, |E| [MHz]**	**p_x,p_y,p_z**	**LW**
$(DPH)_2$				2450, 250	0, 1.0, 0.5	6.0, 0
Me-$(DPH)_3$	2450, 270	0, 1.0, 0	3.0, 0			
$(DPH)_3$	2450, 270	0, 1.0, 0	3.0, 0	3500, 270	0, 1.0, 0.5	4.0, 0

Supplementary Table 2. Simulation parameter for temperature-dependent trEPR spectral simulations (Fig. 3a) using the MATLAB toolbox EasySpin. Parameters given are the ZFS parameters *D* and *E*, relative populations, and the spectral linewidth with Gaussian and Lorentzian contributions [Gaussian Lorentzian]. The populations given for quintet and triplet by SF are the high-field populations, whilst ISC-generated triplet shows zero-field populations.

	**Quintet**			**Triplet by SF**			**Triplet by ISC**		
**T [K]**	**|D|, |E| [MHz]**	**p_{-1},p_0,p_1**	**LW (mT)**	**|D|, |E| [MHz]**	**p_{-1},p_0,p_1**	**LW (mT)**	**|D|, |E| [MHz]**	**p_x,p_y,p_z**	**LW (mT)**
80	750, 80	0, 0.1, 1.0, 0.1, 0	3.0, 0	2450, 270	0, 1.0, 0	3.0, 0		0, 0.8, 1.0	
30	750, 80	0, 0.1, 1.0, 0.1, 0	3.0, 0	2450, 270	0, 1.0, 0	3.0, 0	3900, 300	0, 0.8, 1.0	4.0, 0
10	750, 80	0, 0.1, 1.0, 0.1, 0	3.0, 0	2450, 270	0, 1.0, 0	3.0, 0	3900, 300	0, 1.0, 0.5	4.0, 0

Optically Detected Magnetic Resonance

ODMR is based on difference in PL emission, thus the spectrum is always displayed in an absorptive manner (in contrast to EPR) and no population parameters are used in the simulations. Comparable zero-field splitting parameters as in EPR obtained via a suitable fit. Additionally, the weight of the simulations of quintet and triplet signals are given.

Supplementary Table 3. Simulation parameter for ODMR spectral simulations using the MATLAB toolbox EasySpin. Parameters given are the ZFS parameters *D* and *E*, the spectral linewidth with Gaussian and Lorentzian contributions [Gaussian Lorentzian] and the relative weight between quintet and triplet simulations.

	**Quintet**			**Triplet**		
**Material**	**|D|, |E| [MHz]**	**LW**	**weight**	**|D|, |E| [MHz]**	**LW**	**weight**
$(DPH)_2$	660, 58	10.0, 0	1.0			
Me-$(DPH)_3$	510, 80	8.0, 0	0.3	2400, 290	6.0, 0	0.7
$(DPH)_3$	510, 80	8.0, 0	0.4	2400, 300	8.0, 0	0.6
Me-$(DPH)_3$ at RT	510, 80	8.0, 0	1.0			

Supplementary Table 4. Simulation parameter for ODMR spectral simulations of Me-$(DPH)_3$ at different temperatures using the MATLAB toolbox EasySpin. Parameters given are the ZFS parameters *D* and *E*, the spectral linewidth with Gaussian and Lorentzian contributions [Gaussian Lorentzian] and the relative weight between quintet and triplet simulations. No simulation parameters for 10K given since no ODMR contrast visible.

	**Quintet**			**Triplet**		
**Temp**	**|D|, |E| [MHz]**	**LW**	**weight**	**|D|, |E| [MHz]**	**LW**	**weight**
80K	510, 80	8.0, 0	0.26	2400, 290	6.0, 0	0.74
30K	510, 80	8.0, 0	0.07	2400, 290	6.0, 0	0.93
10K	-	-	-	-	-	-

Supplementary Note 3. Complementarity of TA, trEPR, trPL and ODMR techniques

To measure the full sequence of spin-state formation and emission in singlet fission, we require multiple complementary techniques (Supplementary Table 5). Transient absorption (TA) provides the fastest temporal resolution, directly revealing how and when triplet pairs formed which is important to prove fast triplet pair formation. However, the lack of spin selectivity requires paramagnetic techniques such as electron paramagnetic resonance (EPR). Transient EPR (trEPR) adds spin selectivity, identifying whether triplet and quintet states are occupied. An important advantage of trEPR is the ability to track the populations mechanism of these states via singlet fission or intersystem crossing, but is usually limited to cryogenic temperatures.

Optical detection methods bridge these regimes: transient PL traces delayed fluorescence in high sensitivity up to several hundreds of microseconds. It provides the temporal decay of emissive states as well as the corresponding spectra. However, to identify which paramagnetic species are in the delayed fluorescence, optically-detected magnetic resonance (ODMR) with its spin-selectivity is required. ODMR uniquely identifies paramagnetic species (quintet or triplet) that contribute to luminescence and can be even measured up to room temperature given its good sensitivity.

By combining these complementary approaches, we connect ultrafast population dynamics (TA) with spin-resolved formation pathways (trEPR) and emissive high-spin channels (ODMR), allowing a complete description of the formation and emission of high-spin states in these singlet fission systems. The following table provides and overview about the key information provided by the individual techniques as well as the advantages and limitations.

Supplementary Table 5. Overview of the complementary techniques used to probe spin state formation and emission in these singlet fission systems.

Technique	Key information provided	Advantages / Limitations
**Transient Absorption (TA)**	Tracks formation and decay of all photoexcited states with ps to ns resolution. Identifies fast triplet-pair formation that is unique to singlet fission.	**Advantages:** · Fastest time resolution (ps) · Probes both emissive and non-emissive states · Applicable at room temperature<br><br>**Limitations:** · No intrinsic spin selectivity · Spectral overlap can limit species assignment
**Transient EPR (trEPR)**	Sensitive to spin-polarized paramagnetic states (quintets and triplets). Reveals population mechanism (SF vs ISC) via polarization patterns.	**Advantages:** · Spin-selective: identifies triplet and quintet species · Mechanism-specific information (ISC vs. SF) · Temporal information from 100 ns - μs<br><br>**Limitations:** · Time resolution only from 11-200ns · Typically cryogenic · Lower sensitivity than optical methods
**Transient Photoluminescence (PL)**	Measures radiative decay and delayed fluorescence (ns - μs) with very high sensitivity (performed with ICCD).	**Advantages:** · Directly probes emissive states · Sensitive to delayed emission kinetics and spectra.<br><br>**Limitations:** · Not spin-sensitive (partly with applied magnetic field) · Cannot distinguish triplet vs quintet without ODMR
**Optically Detected Magnetic Resonance (ODMR)**	Monitors PL changes under microwave resonance; identifies directly paramagnetic species (triplet or quintet) that are coupled to luminescence.	**Advantages:** · Very sensitive (lock-in detection) · Spin-selective for emissive paramagnetic states · Usable under continuous excitation and at room temperature<br><br>**Limitations:** · Steady-state technique; no intrinsic time resolution. · Population mechanism inferred indirectly

Supplementary Note 4. Optical characterization of Me-(DPH)$_3$

In the following, we show the optical characterization of the here introduced trimer Me-(DPH)$_3$ by a direct comparison of the steady-state absorption and photoluminescence (PL) spectra, TCSPC and TA of Me-(DPH)$_3$, (DPH)$_3$ and the monomeric DPH.

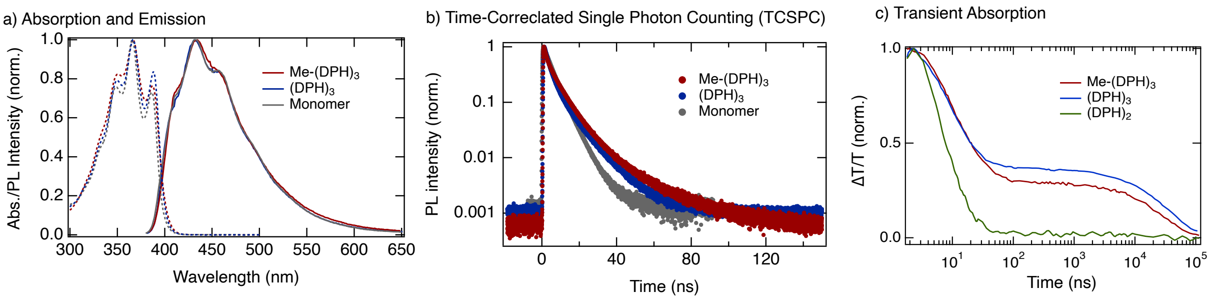

Supplementary Fig. 13. Optical characterisation of Me-(DPH)$_3$. a) Normalized absorption (dashed) and emission (solid) spectra of Me-(DPH)$_3$ (red), (DPH)$_3$ (blue), and DPH monomer (gray). b) TCSPC fluorescence decays showing delayed emission in the trimers due to triplet-pair dynamics, absent in the monomer. c) Nanosecond TA of the TT signal reveals long-lived triplet formation in all oligomers, varying between materials because of different triplet formation via ISC and/or ISC.

Absorption and emission spectra of the Me-substituted trimer closely resemble those of the monomer, and are overall very similar to the unsubstituted (DPH)$_3$ trimer (Supplementary Fig. 13a). However, compared to (DPH)$_3$, Me-(DPH)$_3$ exhibits a modest net blue-shift of the $S_0 \rightarrow S_2$ absorption together with minor vibronic reweighting, consistent with methyl-induced torsion slightly reducing effective conjugation and electronic delocalization. The vibronic structure is otherwise preserved, with a small reduction of vibronic peak contrast and minor broadening, which is consistent with earlier reports of (DPH)$_3$. In the PL spectra, only subtle vibronic redistribution is observed with temperature (see Supplementary Fig. 5), without evidence for additional low-energy shoulders or large spectral shifts. These results suggest that the chromophore units in Me-(DPH)$_3$ maintain their individual electronic character, while methylation perturbs planarity enough to modulate vibronic coupling without fundamentally altering the conjugated backbone. This behaviour mirrors trends in carotenoid polyenes, where $S_0 \rightarrow S_2$ absorption is highly sensitive to conjugation length and vibronic coupling.[2,3] This comparison indicates that the introduction of methyl groups does not radically change the electronic structure relative to the previously studied (DPH)$_3$, but introduces subtle torsional degrees of freedom that are highly relevant for spin-state dynamics.

We also compared the excited-state dynamics of Me-$(DPH)_3$, $(DPH)_3$, and the monomer using TCSPC (Supplementary Fig. 13b). The monomer shows a lifetime of approximately 4.1 ns, consistent with previously reported values for DPH derivatives and reflecting typical S1 decay dynamics. The trimers, in contrast, display multiexponential decays with longer-lived components. This behaviour is characteristic of systems undergoing intramolecular singlet fission, where the emissive S_1 state is coupled to a triplet-pair manifold. The delayed fluorescence observed in both trimers arises from slow re-fusion of weakly-coupled triplet pairs (TT states), including both $^1(TT)$ and $^5(TT)$, back to S_1, which is absent in the monomer. For Me-$(DPH)_3$ and $(DPH)_3$, the TCSPC traces are overall very similar, with only a small offset in the early decay component that we attribute to background counts and measurement uncertainty. Accordingly, we rely on TA as the definitive kinetic comparison. This reinforces that methylation does not significantly affect the early singlet/TT decay kinetics.

To further assess the nature of triplet formation in these systems, we compared the nanosecond TA spectra of Me-$(DPH)_3$, and $(DPH)_3$, as displayed in Supplementary Fig. 13c. As the monomer has no pronounced triplet feature, we show it in comparison with $(DPH)_2$ which enters the TT manifold but has no triplets by singlet fission, instead one triplet per photoexcitation by ISC. This comparison allows us to differentiate between triplet populations arising from singlet fission or ISC.

The dimer $(DPH)_2$, which does not persistently enter the weakly-coupled regime, shows only a small triplet signal plateau, indicative of an inefficient ISC channel. In contrast, both trimers, Me-$(DPH)_3$ and $(DPH)_3$, show clear formation of long-lived triplet features, consistent with triplet-pair generation through SF.
Importantly, while $(DPH)_3$ displays a higher final triplet plateau than Me-$(DPH)_3$, this signal includes contributions from both weakly-coupled and ISC-derived triplets. The Me-substituted trimer, however, exhibits triplet formation solely via singlet fission, with no observable ISC contribution above 80K. This distinction confirms that Me-$(DPH)_3$ serves as a cleaner model system for studying SF-specific dynamics without the complication of parallel ISC pathways - aligning more directly with our design goal of isolating pure SF dynamics.

Supplementary Note 5. Mechanisms of Singlet-Quintet Mixing in Intramolecular Singlet Fission

The conversion of the initially formed singlet multiexciton state ^{1}TT into a quintet state ^{5}TT is central to understanding spin dynamics in singlet fission (SF). This process is governed by the interplay between exchange coupling *J*, molecular geometry, and spin–spin interactions. Two primary mechanisms for singlet–quintet (S-Q) mixing have been proposed: (i) coherent mixing in the low-*J* regime, and (ii) spin polarization transfer under conditions of large but fluctuating *J*.

In the low-*J* mechanism, the exchange interaction becomes transiently small and comparable to the zero-field splitting (ZFS), enabling coherent S-Q mixing. This process leads to spin-polarized quintets, typically favouring $m_s = 0$ sublevels. MacDonald et al. observed such behaviour in pulsed EPR[4], while Matsuda et al. reported similar polarization features in time-resolved EPR of TIPS–pentacene films[5].

In contrast, in the fluctuating high-*J* regime, *J* remains large in magnitude relative to the ZFS but vibrational or conformational dynamics modulate *J*. MacDonald et al. term this the "stochastic" pathway and show with nutation-resolved pulsed EPR that it generates an additional subpopulation of quintets with appreciable $m_s = \pm 2$ occupancy.[4] Matsuda et al. attribute the quintet-derived contribution in disordered TIPS-pentacene films to a similar mechanism via modulation of a large *J* during triplet diffusion and re-encounter, that drives sublevel-selective ^{1}(TT)-^{5}(TT) conversion in amorphous regions.[5] Kobori et al. measure intramolecular pentacene dimers that provide a complementary picture, where terahertz-frequency torsional motions induce *J*-modulation and contribute to singlet-quintet relaxation.[6]

Geometry is therefore central in both regimes. In the low-*J* case, specific conformations or intermolecular arrangements reduce orbital overlap so that *J* becomes comparable to *D*, allowing coherent S-Q mixing or formation of weakly coupled triplet pairs. In the fluctuating high-*J* case, flexible or disordered systems permit rapid torsional and vibrational motions that modulate *J* around a large mean value, enabling non-adiabatic S-Q transitions during these fluctuations.

Supplementary Note 6. Distinguish triplet states generated by singlet fission or ISC

a) trEPR Pattern by Singlet Fission

Triplet states generated by singlet fission can be distinguished from, e.g., SOC-driven ISC, by a special pattern in the trEPR signal. As described in Supplementary Note 5, there are different mixing theories that can lead to the population of the quintet and triplet states. Both mixing scenarios result in a $m_s = 0$ initialized triplet pair state, excluding ISC within the triplet pair spin manifold as this would result in population of zero-field sublevels. The spin polarization on the triplet sublevels is thereby converted from the spin correlated triplet pair states, resulting in overpopulation of $|T_0\rangle$ state in all canonical orientations ($\vec{B} \parallel X, \vec{B} \parallel Y, \vec{B} \parallel Z$). The splitting of the triplet sublevels thereby depends on the orientation of the external magnetic field $\vec{B}$ with respect to the principal axes X, Y, and Z of the ZFS tensor.[7] The energy of the aligned state, called canonical orientation, is thus independent of B and equal to the energy at zero field, while the energies of the other two states increase/decrease with magnetic field (Supplementary Fig. 14). The overpopulated $|T_0\rangle$ state thus leads to an *eaaeea* or *aeeaae* pattern as shown in Supplementary Fig. 14. This pattern can only be obtained by selective population of high-field spin states and can thus be distinguished from triplet excitons occupied by SOC-driven ISC.

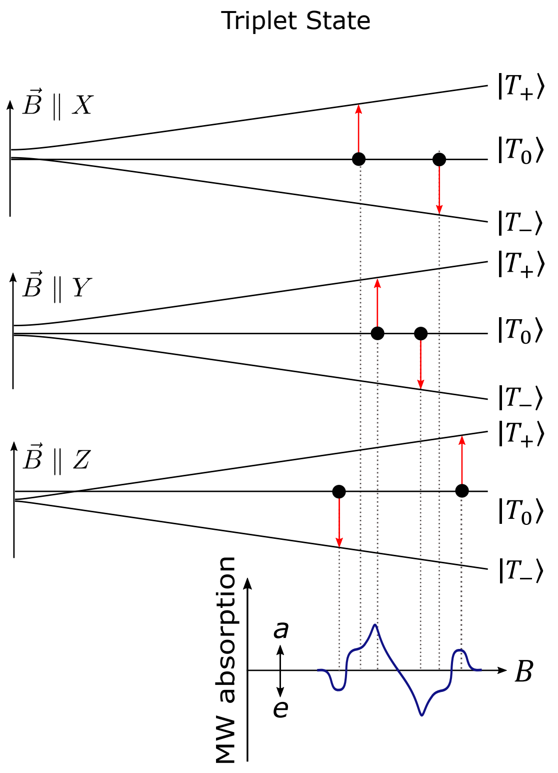

Supplementary Fig. 14. Generation of a trEPR spectrum of triplets generated by singlet fission. Due to angular momentum conservation, spin mixing occurs between $|^1(TT)\rangle$ and $|^5(TT)_0\rangle$ ($m_s = 0$). Dissociation into free triplet excitons preserves spin polarization, whereby $|T_0\rangle$ states get overpopulated, arising here in an *eaaeea* pattern.

b) trEPR Pattern by SOC-driven ISC

SOC is the dominant mechanism for ISC in small molecules and decays rapidly with electron-hole distance with typical coupling values of few meV. The trEPR pattern arising from ISC-born triplets in this manuscript possess zero-field populations of $[p_x, p_y, p_z] = [0, 1.00, 0.5]$. SOC-driven ISC acts on the zero-field triplet states, given by the eigenstates of the Hamiltonian $|X\rangle$, $|Y\rangle$ and $|Z\rangle$, whose populations can be transferred to the populations of the high-field states $|T_+\rangle$, $|T_0\rangle$, $|T_-\rangle$. The population of the high-field state is therefore different for each canonical orientation:[8]

$$\vec{B}||Z: p_0 = p_z, p_\pm = 0.5\,(p_x + p_y)$$

$$\vec{B}||Y: p_0 = p_y, p_\pm = 0.5\,(p_x + p_z)$$

$$\vec{B}||X: p_0 = p_x, p_\pm = 0.5\,(p_y + p_z)$$

Since for $\vec{B}||Z$, p_0 and $p_\pm$ are almost equal, this transition is minor pronounced in the shown trEPR spectra. For each orientation of the principal axis of D with respect to $\vec{B}$, there are enhanced absorptive and emissive transitions in magnetic resonant conditions (Supplementary Fig. 15), which add up in rigid samples to a characteristic powder pattern:

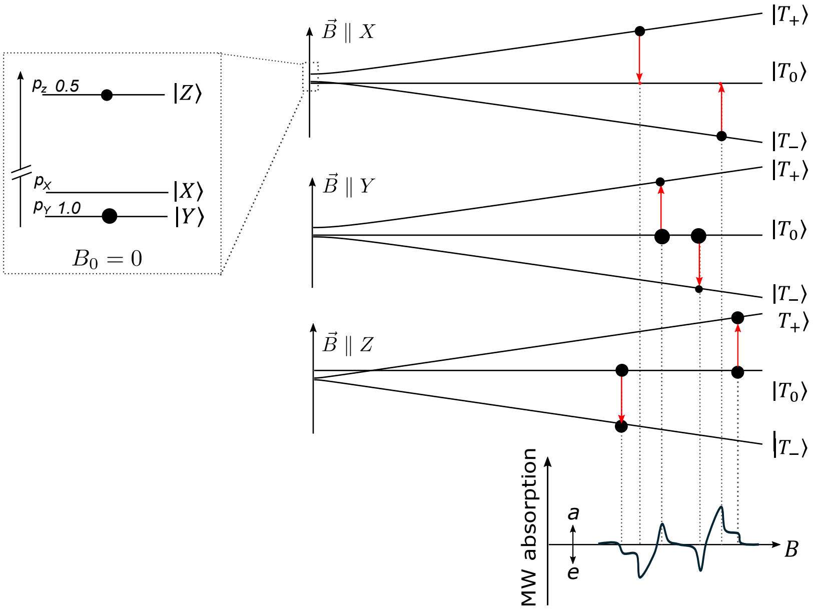

Supplementary Fig. 15. Generation of a SOC-generated triplet trEPR spectrum with $[p_x, p_y, p_z] = [0, 1.0, 0.5]$. ISC is sublevel-selective and acts on the zero-field sublevels $|X\rangle$, $|Y\rangle$ and $|Z\rangle$. These populations are converted into high-field populations $|T_+\rangle$, $|T_0\rangle$ and $|T_-\rangle$, depending on the principal

axes (X, Y, Z) orientation of the ZFS tensor D with respect to magnetic field $\vec{B}$. The unequal populations result in microwave emission (e) or absorption (a) for $\Delta m_s = \pm 1$ transitions (red arrows) for each canonical orientation. The spectra of all orientations of D relative to $\vec{B}$ add up to a characteristic trEPR signal (blue).

Supplementary Note 7. Influence of Triplet-Triplet-Annihilation (TTA)

The ODMR triplet features observed in the trimers at low temperature can be consistently explained by weakly coupled triplet pairs that persist long enough to undergo geminate triplet–triplet annihilation (TTA). At cryogenic temperatures, these states remain sufficiently long-lived and spin-polarized that microwave-driven transitions between the triplet sublevels alter the fraction of recombination events feeding back into the singlet manifold, giving rise to the characteristic triplet ODMR signatures. This mechanism is well established in both singlet-fission chromophores and OPV materials at cryogenic temperatures.[9,10] The absence of any triplet ODMR feature in the dimer, which does not access a persistent weakly coupled regime, further supports this assignment to geminate TTA from (T…T) states.

At elevated temperatures, however, the residence time of these weakly coupled triplet pairs becomes too short to maintain detectable spin polarization within the ODMR detection window. Faster spin relaxation and more rapid recombination suppress the visibility of the triplet channel, leaving ^{5}TT as the only long-lived high-spin state observed at room temperature. This temperature-dependent suppression of TTA resonances is a general trend in ODMR studies of singlet fission, where triplet encounters are only detectable at cryogenic temperatures.

We emphasize that while TTA contributes to the ODMR signal at low temperature, our data do not support it as a dominant emissive pathway under ambient conditions. All three oligomers, including the dimer that cannot undergo geminate TTA, show nearly identical trPL decay kinetics at room temperature, with the lifetime comparable to the ^{5}TT states, and ODMR spectra display only quintet emission. Thus, although TTA and associated ^{3}(T…T) intermediates play a role at low temperatures, quintet-mediated emission dominates under operating conditions relevant for room-temperature singlet fission.

Supplementary Note 8. Magnetic field Effect

High-Field MFEs

At magnetic fields above ~100 mT, we observe a notable enhancement in PL from the DPH trimers. This behaviour is consistent with magneto-optical effects reported in tetracene trimers as well as DPH films.[11,12] The observed PL enhancement at high magnetic fields can be attributed to field-induced suppression of spin mixing between correlated triplet pair states, specifically the conversion from singlet-paired ^{1}(T…T) to quintet-paired ^{5}(T…T). As the external magnetic field increases, Zeeman splitting stabilizes the m_s sublevels and reduces spin interconversion, which suppresses the generation of dissociated triplets. This is supported by our TA measurements, which show a decrease in the triplet signal amplitude with increasing magnetic field.

The reduced efficiency of SF under high fields preserves more singlet excitons, which in turn increases the population available for radiative decay via S_1 or ^{1}TT, resulting in enhanced PL. This mechanism closely mirrors that seen in previously studied tetracene oligomers and intermolecular DPH systems, where magnetic fields suppress triplet formation and reroute exciton decay through emissive singlet channels.

Low-Field MFEs and Exchange Interaction Regimes

In our DPH systems, we observe a negative MFE below ~50 mT, which transitions into a positive MFE at higher fields, with a zero-crossing around 50 mT. At zero field, the singlet character is confined to only three TT spin states. As the magnetic field is increased into the low-field regime (<50 mT), the number of spin states that carry singlet character increases up to a maximum of six (for identical triplets). This redistribution suppresses recombination into emissive singlet products, giving rise to the negative fluorescence MFE observed at low fields. At higher fields (>50 mT), as the Zeeman interaction dominates over the weak exchange coupling, the singlet character becomes restricted to just two states. In this regime, spin mixing is reduced and recombination pathways feeding back into the singlet state are enhanced, leading to the positive MFE component.

This redistribution framework explains the negative-to-positive lineshape observed in Fig. 2d, and matches the canonical description of low-field effects in triplet–triplet pairs.

Supplementary Note 9. Spin States in DPH Systems

The wavefunctions for ^{5}TT, with each 4 spins, reading at high magnetic field values as follows:[13]

$|^5(\mathrm{TT})_2\rangle = |2,\ 2\rangle = |+\,+\rangle$

$|^5(\mathrm{TT})_1\rangle = |2,\ -1\rangle = 1/\sqrt{2}(|{+0}\rangle\ - |0\,+\rangle)$

$|^5(\mathrm{TT})_0\rangle = |2,\ 0\rangle = 1/\sqrt{6}(2|00\rangle + |+\,-\rangle + |+\,-\rangle)$

$|^5(\mathrm{TT})_{-1}\rangle = |2,\ -1\rangle = 1/\sqrt{2}(|{-0}\rangle + |0\,-\rangle)$

$|^5(\mathrm{TT})_{-2}\rangle = |2,\ -2\rangle = |-\,-\rangle$

With $|+\rangle = |\uparrow\uparrow\rangle$, $|0\rangle = |\uparrow\downarrow\rangle$ and $|-\rangle = |\downarrow\downarrow\rangle$.

For the singlet state, we denoted the initial singlet state as S*, with S* being denoted as the singlet state in DPH literature, based on rapidly equilibration of the S_1 and S_2 states on sub-picosecond timescales.[14] We also consider that singlet fission literature uses the terminology 1Bu and 2Ag, where 2Ag describes the lowest-lysing singlet state, which would be ^{1}TT in our systems.[15] However, to be still consistent with literature in spin physics and spin chemistry, we denoted the singlet states with triplet pair character, displaying a 4-spin state, as ^{1}TT.

Depending on the system, there is evidence in longer systems like DPO or oligoene systems of ^{1}TT emission. However, the DPH display shorter systems and the following experimental observations let us believe the S* being the emissive state: (1) The spectrum of the monomer to the dimer and trimer are overlapping, meaning the 2Ag/^{1}TT state would be a state also present in the monomeric version. (2) The photoluminescence quantum yield of the monomer is at 73%, meaning this 2Ag state would be a very bright state. (3) The temperature activation of PL but the possibility of formation of quintet and triplet states at all temperatures would require a conformational temperature activation to the emissive 1TT state that would only influence the back pathway, but not the forward pathway. (4) We discussed before in our DPH systems that PL spectra of all presented materials, including monomer pTol-DPH, are nearly indistinguishable.[16] This indicates that the PL of these materials are all identical to the PL of the monomeric reference compound, indicating that emission arises from the single chromophore S* state.

Supplementary Note 10. Kinetic Model

In the following, we introduce a model to obtain k_{SF}, k_{revSF} and k_S. Our work presented resolves a broad set of spin states – including ^{1}(TT), ^{5}(TT), ^{n}(T···T), and both SF- and ISC-generated triplets. This increased mechanistic resolution introduces a significantly larger number of kinetic parameters in the model, which makes the solution of a complete kinetic model not trustworthy. In order to keep the model solvable and well-constrained, we focused for the calculated parameters on the early-time regime (<50 ns), where dissociation of TT into long-lived free triplets (T...T) and subsequent high-spin states, as quintet states ^{5}(TT), is negligible. However, to give a complete picture, Supplementary Fig. 17 gives all present rates in the systems, timescales of high-spin states as well as displays the optical back pathway for ^{5}(TT).

We constructed a kinetic model based on first-order differential equations describing reversible singlet fission between the S_1 state and the correlated triplet-pair state (TT):

$$\frac{dS_1}{dt} = -k_{SF} \times S_1 + k_{revSF} \times {}^1TT - k_S \times S_1$$

$$\frac{d\,{}^1TT}{dt} = +k_{SF} \times S_1 - k_{revSF} \times {}^1TT$$

From psTA and nsTA (Supplementary Fig. 16), we obtained k_{SF} = 2.8x10^{10} s^{-1} and k_{revSF} = 8.2x10^7 s^{-1}. A biexponential fit to the TCSPC decay yields k_1 = 6.3x10^8 s^{-1} and k_2 = 1.0x10^8 s^{-1}. Whilst k_{SF} lies within the response time of the setup, k_1 corresponds well to the direct singlet decay k_S, whilst the slower component likely arises from reverse SF, repopulating S_1, consistent with the independently determined k_{revSF} rate.

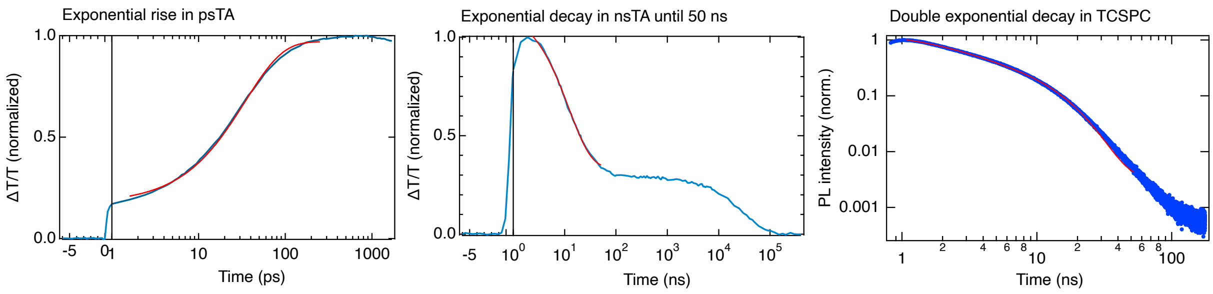

Supplementary Fig. 16. Kinetic model for (Me-DPH)$_3$. From the exponential rise of the TT states in the psTA data, we can obtain the k_{SF} = 2.8x10^{10} s^{-1}, whilst nsTA obtains a reverse SF rate of k_{revSF} = 8.2x10^7 s^{-1}. TCSPC data yields k_1 = 6.3x10^8 s^{-1} and k_2 = 1.0x10^8 s^{-1}.

To quantify the delayed fluorescence contribution associated with quintet emission, we compare time-resolved and magnetic-field-dependent PL data. The magnetic field response appears within ~40 ns after excitation, indicating that the singlet population has already decayed and the delayed PL originates from high-spin states. Integration of the PL signal beyond 40 ns shows that this delayed component accounts for approximately 10% of the total emission until 2µs. This timescale is in agreement with the decay of the ^{5}TT population observed in trEPR. The trPL is comparable for all three compounds at room temperature, including the dimer that does not enter the weakly-coupled regime and only has emission via quintets. Thus, these quantitative comparisons substantiate our conclusion that quintet-state emission dominates the delayed fluorescence channel in about 10% of the total emission. Supplementary Fig. 17 shows the optical back-pathway of these ^{5}TT states via the spin-allowed back pathway $^5TT \rightarrow {}^1TT \rightarrow S_1$.

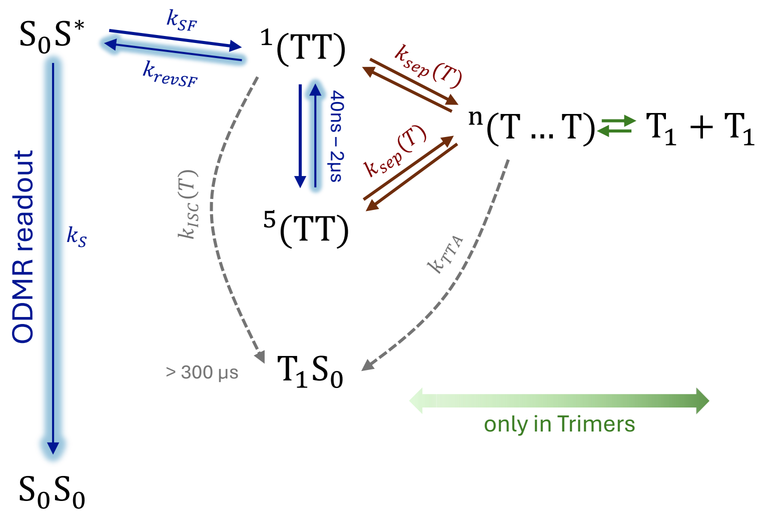

Supplementary Fig. 17. Kinetics and rates in studies DPH oligomers. k_S, k_{SF} and k_{revSF} obtained through kinetic model, k_{ISC} temperature dependent for Me-(DPH)$_3$ and present at all temperatures for (DPH)$_2$ and (DPH)$_3$. In trimers, separation forms ^{n}(T…T) states which undergo an equilibrium with ^{1}TT and ^{5}TT or undergo k_{TTA} to T_1S_0. Glowed blue arrows display the optical back pathway for ^{5}(TT), which participates in luminescence up to 2µs.

Supplementary Note 11. Comparison to other Singlet Fission Systems

To position the investigated DPH oligomers within the SF landscape, we compare in the following the DPH oligomers to previous dimer and trimer works on tetracene and perylene dimers.

Work on tetracene oligomers has shown that increasing the number of chromophore units enables access to spatially separated, weakly coupled triplet pairs ^{1}(T…T), which enhances free-triplet generation compared with dimers that primarily populate adjacent ^{1}TT states.[12] This established a structural modulation, based on chromophore number and geometry, for the degree of intertriplet coupling and triplet separation. Our DPH oligomers are consistent with this picture where trimers can access a weakly coupled (T…T) manifold at low temperature, as seen in ODMR and trEPR, whereas the dimer does not enter it persistently. However, our study goes beyond triplet-yield optimization by directly resolving and exploiting the quintet ^{5}TT manifold: we observe ^{5}TT formation in all oligomers and quintet-mediated delayed fluorescence up to room temperature, detected by ODMR.

Similar studies on perylene dimers highlights that low-frequency conformational motions and interchromophore coupling influences access to distinct multiexciton manifolds.[17] Consistent with that principle, our DPH oligomers show that methylation modulates low-frequency torsions, biasing triplet-pair mixing relative to ISC. However, our scope and outcome differ: prior perylene/tetracene studies optimized free-triplet yields but did not directly address quintet formation or emission. Here, we identify the ^{5}TT manifold spectroscopically and show that it feeds delayed fluorescence via spin-allowed back-transfer the emissive singlet state. To our knowledge, room-temperature optical detection of emissive quintets acting as an emissive reservoir has not been reported for tetracene or perylene oligomers.

However, also in terms of triplet formation for singlet fission application, DPH offers an important advantage: the DPH oligomers have a triplet energy of ~1.5 eV, well above the silicon bandgap and higher than that of tetracene (~1.25 eV) or perylene (~1.1 eV), making it a promising singlet-fission material for silicon solar cells. Thus, DPH oligomers bridge the gap between energy-conversion systems and quantum-applications, establishing a new regime where high-spin states are not only intermediates but functional emissive states that can be used towards molecular quantum-optical applications.

Supplementary Note 12. Synthetic Details

The synthetic procedures and NMR spectra for $(DPH)_2$ and $(DPH)_3$ are reproduced from our previous work[16] for completeness and direct comparison with the newly synthesized Me-$(DPH)_3$. These compounds were synthesized in the same way as in the previous reports in our laboratory, thus the synthetic procedures for $(DPH)_2$ and $(DPH)_3$ are unchanged from our earlier report. Their inclusion here allows for a side-by-side evaluation with the new derivative Me-$(DPH)_3$.

General Synthesis and Characterisation Details

Glassware was routinely dried in an oven set to 200 °C before use. Flash column chromatography purifications were performed on an autocolumn (Biotage® Isolera) using pre-packed silica gel cartridges purchased from Biotage®. Column conditions were obtained by analytical thin layer chromatography (TLC) using precoated glass backed silica gel plates (Merck Kieselgel 60 F254 0.20 mm). Visualisation was achieved throughout using UV light (254, 365 nm). Nuclear magnetic resonance spectroscopy was performed on a Bruker 400 MHz Avance III HD Spectrometer, a Bruker 500 MHz Avance III HD Spectrometer or a Bruker 700 MHz TXO Cryoprobe Spectrometer. All spectra taken on these machines were recorded at ambient temperature in the stated solvent with residual protic solvent used as the internal standard. 1H NMR data is reported as: chemical shift (multiplicity, coupling constant, integration). 13C NMR data is reported as: chemical shift. Chemical shifts (δ) are quoted in parts per million (ppm) relative to residual solvent (CDCl3: δ(1H) = 7.26 ppm, δ(13C) = 77.16 ppm); Standard abbreviations are used to indicate multiplicities and peak forms: s = singlet, d = doublet, t = triplet, q = quartet, p = pentet, sx = sextet, sp = septet, m = multiplet, br = broad and associated combinations thereof. Coupling constants (J) are quoted to the nearest 0.1 Hz and are assumed to be JH-H unless otherwise stated. Mass spectra were obtained using a Waters LCT, Finnigan MAT 900XP or Waters MALDI micro MX spectrometer by the mass spec team at the Department of Chemistry, University of Cambridge.

Chemicals

All commercial chemicals were of ≥95% purity and were used as received without further purification. Anhydrous solvents were purchased from Sigma Aldrich or Acros Organics and used as received for synthesis. Once synthesized, final DPH materials were stored under argon at ~4 °C or in a nitrogen glovebox at ambient temperature. Tetraethyl but-2-ene-1,4-diyl(E)-bis(phosphonate)[18], 4-(2-ethylhexyl)benzaldehyde[19], (1E,3E,5E)-1,6-bis(3-bromophenyl)hexa-1,3,5-triene **[4]**[3] and 1-bromo-3-((1E,3E,5E)-6-(4-(2-ethylhexyl)phenyl)hexa-1,3,5-trien-1-yl)benzene **[1]**[2] were prepared as previously reported.

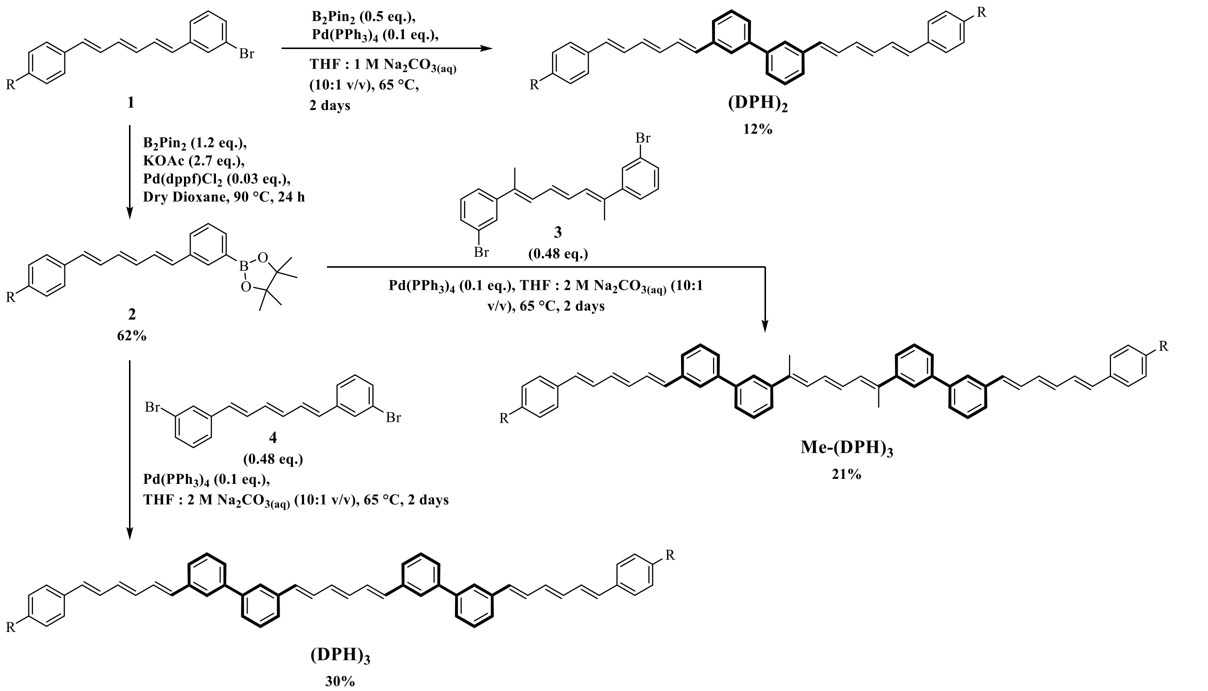

Supplementary Fig. 18. Synthesis of $(DPH)_2$, $(DPH)_3$ and Me-$(DPH)_3$. Reaction scheme outlining the synthesis of $(DPH)_2$, $(DPH)_3$ and Me-$(DPH)_3$ as described below.

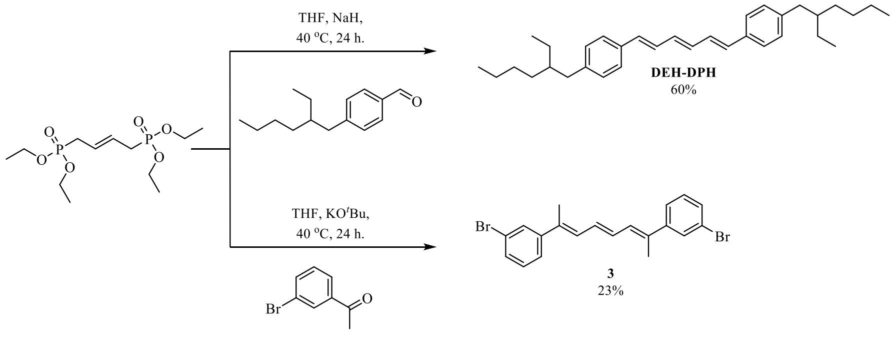

Supplementary Fig. 19. Synthesis of DEH-DPH and 3,3'-((2E,4E,6E)-octa-2,4,6-triene-2,7-diyl)bis(bromobenzene). Reaction scheme outlining the synthesis of DEH-DPH and 3,3'-((2E,4E,6E)-octa-2,4,6-triene-2,7-diyl)bis(bromobenzene) [3].

3,3'-bis((1E,3E,5E)-6-(4-(2-ethylhexyl)phenyl)hexa-1,3,5-trien-1-yl)-1,1'-biphenyl [$(DPH)_2$]

A microwave vial was charged with a stir bar, **1** (100 mg, 0.24 mmol, 1.0 eq.), bis(pinacolato)diboron (30 mg, 0.12 mmol, 0.5 eq.), and $Pd(PPh_3)_4$ (27 mg, 0.024 mmol, 0.1 eq.) and flushed under argon. THF (7 mL) and an aqueous solution of Na_2CO_3 (1M, 0.7 mL) were separately degassed and then added to the solid reagents. The reaction mixture was lowered into a preheated oil bath and heated at 65 °C in the dark for 2 days. The reaction mixture was carefully acidified with 1 M HCl (~ 3 mL) with stirring and then diluted with DCM (~75 mL) and brine (~50 mL). The organic layer was separated and washed with brine (~ 20 mL), dried ($MgSO_4$) and the solvent remove *in vacuo*. The crude material was then purified by flash column chromatography (eluent: DCM/ *n*-hexane gradient from 0:100 → 10:0 v/v).

Following removal of the solvent the columned material was sonicated in hexane to obtain the product, **(DPH)₂**, as an off-white powder (20 mg, 12%).

^{1}H NMR (500 MHz, $CDCl_3$) δ 7.65 (s, 2H), 7.48 – 7.42 (m, 6H), 7.36 (d, *J* = 8.2 Hz, 4H), 7.13 (d, *J* = 8.2 Hz, 4H), 7.02 – 6.97 (m, 2H), 6.92 – 6.87 (m, 2H), 6.68 (d, *J* = 15.5 Hz, 2H), 6.62 (d, *J* = 15.4 Hz, 2H), 6.59 – 6.52 (m, 4H), 2.56 – 2.48 (m, 4H), 1.58 – 1.53 (m, 2H), 1.33 – 1.24 (m, 16H), 0.90 – 0.86 (m, 12H).

13C NMR (176 MHz, CD_2Cl_2) δ 142.3, 141.8, 138.4, 135.1, 134.6, 133.4, 133.3, 132.5, 130.1, 130.0, 129.5, 128.5, 126.7, 126.5, 125.7, 125.5, 41.5, 40.2, 32.7, 29.2, 25.8, 23.5, 14.3, 11.0.

HRMS(m/z) Found [M+H]+ = 687.4915, $C_{52}H_{62}$ requires 687.4930, Δ = -1.5 ppm

2-(3-((1E,3E,5E)-6-(4-(2-ethylhexyl)phenyl)hexa-1,3,5-trien-1-yl)phenyl)-4,4,5,5-tetramethyl-1,3,2-dioxaborolane [2]

2 was prepared by a Miyaura borylation from the bromide **1**. **1** (846 mg, 2.0 mmol, 1.0 eq.), bis(pinacolato)diboron (609 mg, 2.4 mmol, 1.2 eq.), Pd(dppf)Cl_2 (44 mg, 0.06 mmol, 0.03 eq.), potassium acetate (530 mg, 5.4 mmol, 2.7 eq.) and stir bar were added to a Schlenk flask, which was evacuated and backfilled with argon 5 times. Dry dioxane (40 mL) was added, the reaction mixture was lowered into a preheated oil bath and stirred at 90 °C in the dark for 24 h. The reaction mixture was allowed to cool to room temperature and then the solvent removed *in vacuo*. The crude material was dissolved in DCM, dry loaded onto silica and purified by flash column chromatography (eluent: DCM/ *n*-hexane gradient from 0:100 → 30:70 v/v). After removal of the solvent, the material was sonicated in MeOH, filtered and dried under suction to obtain the product, **2**, as an off-white powder (580 mg, 62%).

^{1}H NMR (500 MHz, $CDCl_3$) δ 7.86 (s, 1H), 7.66 (d, J = 7.3 Hz, 1H), 7.50 (d, J = 7.8 Hz, 1H), 7.34 – 7.32 (m, 3H), 7.10 (d, J = 7.7 Hz, 2H), 6.96 – 6.91 (m, 1H), 6.87 – 6.82 (m, 1H), 6.61 – 6.46 (m, 4H), 2.58 – 2.50 (m, 2H), 1.61 – 1.54 (m, 1H), 1.36 (s, 12H), 1.34 – 1.26 (m, 8H), 0.91 – 0.88 (m, 6H).

^{13}C NMR (126 MHz, $CDCl_3$): δ 141.8, 137.0, 134.9, 134.0, 133.9, 133.3, 132.9, 132.9, 132.4, 129.7, 129.5, 129.3, 128.4, 128.2, 126.3, 84.0, 41.2, 40.1, 32.5, 29.0, 25.6, 25.0, 23.2, 14.3, 10.9.

HRMS(m/z) Found [M+H]+ = 471.3429, $C_{32}H_{43}BO_2$ requires 471.3434, Δ = -0.5 ppm

(1E,3E,5E)-1,6-bis(3'-((1E,3E,5E)-6-(4-(2-ethylhexyl)phenyl)hexa-1,3,5-trien-1-yl)-[1,1'-biphenyl]-3-yl)hexa-1,3,5-triene [(DPH)₃]

A 10 mL microwave vial was charged with a stir bar, **2** (282 mg, 0.60 mmol, 2.1 eq.), **4** (111 mg, 0.29 mmol, 1.0 eq.), and Pd(PPh_3)$_4$ (35 mg, 0.03 mmol, 0.1 eq.) and flushed under argon. THF (4 mL) and an aqueous solution of Na_2CO_3 (2M, 0.9 mL) were separately degassed and then added to the solid

reagents. The reaction mixture was lowered into a preheated oil bath and heated at 65 °C in the dark for 2 days. The reaction mixture was carefully acidified with 1 M HCl (~ 4 mL) with stirring and then diluted with DCM (~100 mL) and brine (~75 mL). The organic layer was separated and washed with brine (~ 30 mL), dried ($MgSO_4$) and the solvent remove *in vacuo*. The crude material was then purified by flash column chromatography (eluent: DCM/ *n*-hexane gradient from 0:100 → 35:70 v/v). Following removal of the solvent from the fractions containing only the product (identified by TLC) the material was sonicated in MeOH and the solid filtered to obtain **(DPH)₃** as a pale yellow powder (83 mg, 31%).

^{1}H NMR (500 MHz, $CDCl_3$) δ 7.64 (m, 4H), 7.47 – 7.40 (m, 12H), 7.34 (d, *J* = 8.2 Hz, 4H), 7.11 (d, *J* = 8.2 Hz, 4H), 7.02 – 6.95 (m, 4H), 6.89 – 6.85 (m, 2H), 6.71 – 6.50 (m, 12H), 2.55 – 2.49 (m, 4H), 1.58 (m, 2H), 1.35 – 1.23 (m, 16H), 0.91 – 0.86 (m, 12H).

^{13}C NMR (126 MHz, $CDCl_3$): δ 141.7, 141.6, 138.0, 137.9, 134.7, 134.2, 133.8, 133.0, 132.9, 132.7, 132.1, 129.7, 129.5, 129.1, 128.2, 126.5, 126.4, 126.2, 125.4, 125.3, 125.3, 125.2, 41.2, 40.1, 32.5, 29.0, 25.6, 23.2, 14.3, 10.9.

HRMS(m/z) Found [M+H]+ = 917.5987, $C_{70}H_{76}$ requires 917.6025, Δ = -3.8 ppm

3,3'-((2E,4E,6E)-octa-2,4,6-triene-2,7-diyl)bis(bromobenzene) [3]

To an oven-dried flask under argon was added tetraethyl but-2-ene-1,4-diyl(E)-bis(phosphonate) (2.00 g, 6.09 mmol, 1 equiv.) and 3-bromo-acetophenone (2.67 g, 13.4 mmol, 2.2 equiv.) in anh. THF (70 mL) was added a freshly prepared solution of potassium tert butoxide (2.05 g, 18.3 mmol, 3 equiv.) in anhydrous THF (30 mL) dropwise in the dark. The reaction was then heated to 40 ºC and left to stir for 24 h. Water (equal vol. to THF) was added, and the mixture stirred for 1 h. The mixture was extracted with DCM (5×). The combined organic extracts were washed with water (2×), brine (2×), dried over MgSO4 as concentrated in vacuo. The crude oil was purified by column chromatography (Hexane: DCM, 9:1). After evaporation of the column solvent the resulting solid was sonicated in methanol and then isolated via filtration to obtain **3** as a yellow crystalline solid (586 mg, 23%).

^{1}H NMR (400 MHz, $CDCl_3$) δ 7.62 (t, *J* = 1.9 Hz, 2H), 7.43 – 7.33 (m, 4H), 7.21 (t, *J* = 7.9 Hz, 2H), 6.75 (m, 2H), 6.67 – 6.57 (m, 2H), 2.19 (d, *J* = 1.1 Hz, 6H).

^{13}C NMR (101 MHz, $CDCl_3$): δ 145.2, 135.4, 130.8, 130.1, 129.9, 128.8, 128.6, 124.3, 122.8, 16.1.

HRMS(m/z) Found [M+H]+ = 415.9771, $C_{20}H_{19}Br_2$ requires 415.9775, Δ = 1.0 ppm

3',3'''-((2E,4E,6E)-octa-2,4,6-triene-2,7-diyl)bis(3-((1E,3E,5E)-6-(4-(2-ethylhexyl)phenyl)hexa-1,3,5-trien-1-yl)-1'',1'''-biphenyl) [Me-(DPH)₃]

Me-(DPH)₃ was prepared by an analogous Suzuki coupling procedure to the preparation of **(DPH)₃** on the same scale using: **2** (282 mg, 0.60 mmol, 2.1 eq.), **3** (120 mg, 0.29 mmol, 1.0 eq.) $Pd(PPh_3)_4$ (35 mg,

0.03 mmol, 0.1 eq.), THF (4 mL), and an aqueous solution of Na_2CO_3 (2M, 0.9 mL). Following the work-up and purification steps **Me-(DPH)₃** was obtained as a yellow powder (58 mg, 21%).

^{1}H NMR (500 MHz, $CDCl_3$) δ 7.70 (s, 2H), 7.64 (s, 2H), 7.52 – 7.37 (m, 12H), 7.33 (d, *J* = 8.1 Hz, 4H), 7.11 (d, *J* = 8.1 Hz, 4H), 7.01 – 6.93 (m, 2H), 6.91 – 6.72 (m, 6H), 6.67 (d, *J* = 15.5 Hz, 2H), 6.62 – 6.48 (m, 6H), 2.57 – 2.46 (m, 4H), 1.57 (s, 2H), 1.35 – 1.22 (m, 16H), 0.87 (m, 12H).

^{13}C NMR (176 MHz, $CDCl_3$): δ 143.7, 142.0, 141.9, 141.4, 138.1, 136.4, 134.9, 134.3, 133.1, 133.0, 132.3, 130.6, 129.8, 129.7, 129.2, 128.9, 128.3, 128.1, 126.6, 126.3, 126.2, 125.5, 125.4, 124.8, 124.7, 41.2, 40.1, 32.5, 29.0, 25.6, 23.2, 16.4, 14.3, 10.9.

HRMS(m/z) Found [M+H]+ = 945.6321 $C_{72}H_{81}$ requires 945.6338, Δ = -1.8 ppm

(1E,3E,5E)-1,6-Bis(4-(2-ethylhexyl)phenyl)hexa-1,3,5-triene [DEH-DPH]

To an oven-dried flask under argon was added tetraethyl but-2-ene-1,4-diyl(E)-bis(phosphonate) (0.54 g, 1.65 mmol, 1 equiv.) and anh. THF (10 mL). Sodium hydride (0.080 g, 3.96 mmol, 1.2 equiv.) was then added, followed by some more anh. THF (5 mL), and the resulting mixture was allowed to stir at RT for 30 mins (cloudy grey mixture). To this, a pre-dissolved solution of 4-(2-ethylhexyl)benzaldehyde (1.0 g, 4.12 mmol, 2.5 equiv.) in anh. THF (10 mL), was added dropwise over 5 mins and then left to stir for 30 mins at RT. A second dose of sodium hydride (0.080 g, 3.96 mmol, 1.2 equiv.) was added, followed by anh. THF (5 mL), which was then heated to 40 °C and left to stir for 24 h. The resulting emulsion (pale yellow solution and golden orange oily layer) was then hydrolysed with ice water (60 mL), forming a yellow solution. The mixture was extracted with diethyl ether and the organic phases were separated and dried over $MgSO_4$, filtered through cotton wool, and concentrated *in vacuo*. The yellow oil was dry loaded onto silica and purified *via* column chromatography (Hexane). The fractions containing product were combined and concentrated *in vacuo,* and then sonicated in methanol to give **DEH-DPH** as pale-yellow powder (0.45 g, 60%).

^{1}H NMR (600 MHz, $CDCl_3$) δ 7.32 (d, *J* = 8.2 Hz, 4H, Ph*H*), 7.10 (d, *J* = 8.2 Hz, 4H, Ph*H*), 6.85 (ddd, *J* = 15.5, 7.0, 3.1 Hz, 2H, C*H*=CH), 6.57 (d, *J* = 15.5 Hz, 2H, C*H*=CH), 6.53 – 6.45 (m, 2H, C*H*=CH), 2.59 – 2.46 (m, 4H, CH_2CH), 1.56 (m, 2H, CH_2C*H*), 1.35 – 1.18 (m, 16H, C$H_2$$CH_3$), 0.88 (m, 12H, C$H_3$).

^{13}C NMR (151 MHz, $CDCl_3$): δ 141.5, 134.8, 133.2, 132.4, 129.5, 128.3, 126.1, 41.1, 39.9, 32.3, 28.8, 25.4, 23.0, 14.1, 10.8.

HRMS(m/z) Found [M+H]+ = 456.3729, $C_{34}H_{48}$ requires 456.3751, Δ = -2.2 ppm

NMR Spectra

3,3'-bis((1E,3E,5E)-6-(4-(2-ethylhexyl)phenyl)hexa-1,3,5-trien-1-yl)-1,1'-biphenyl [(DPH)$_2$]

^{1}H NMR, 500 MHz, $CDCl_3$

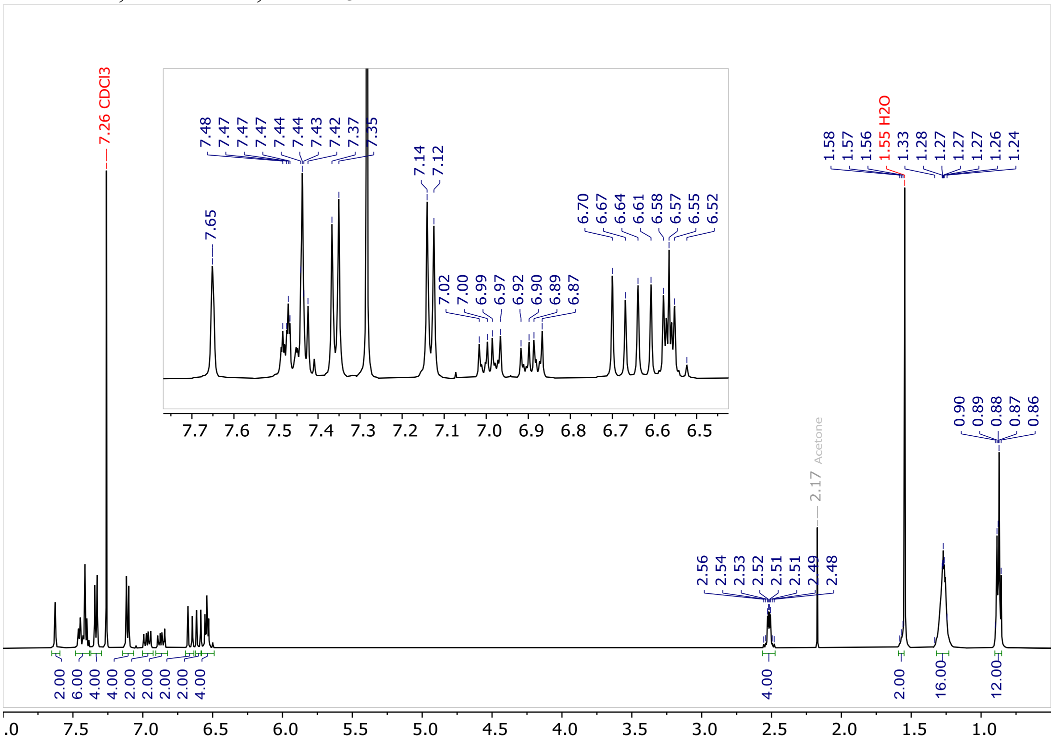

^{13}C NMR, 176 MHz, CD_2Cl_2

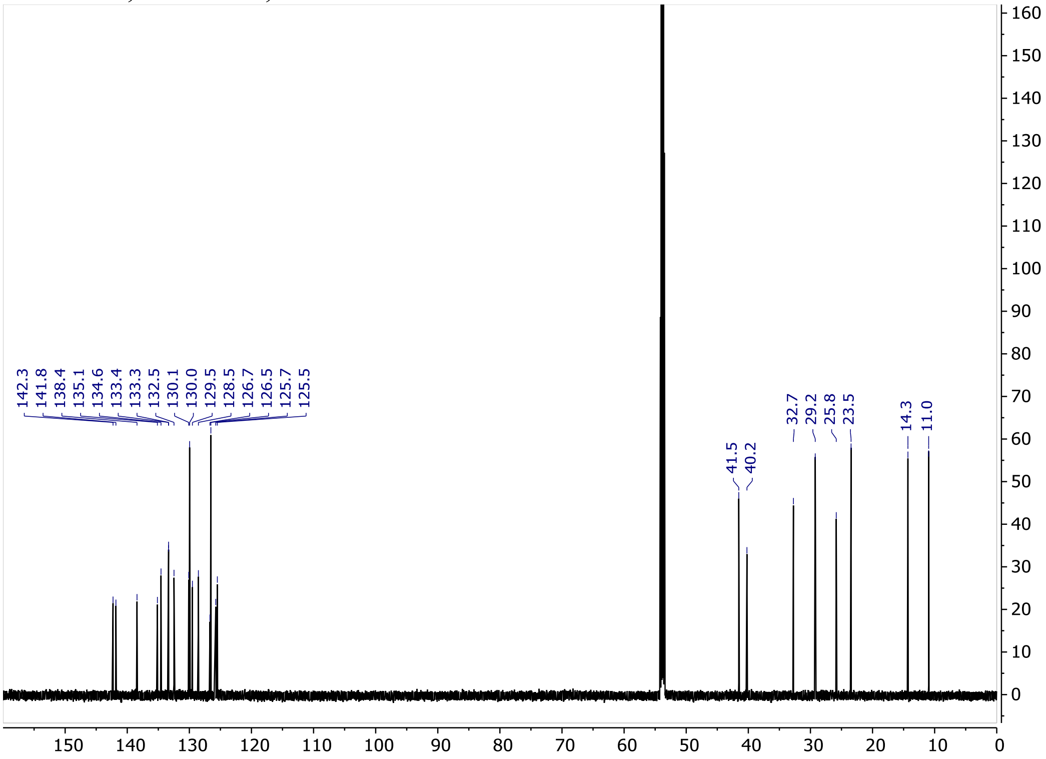

2-(3-((1E,3E,5E)-6-(4-(2-ethylhexyl)phenyl)hexa-1,3,5-trien-1-yl)phenyl)-4,4,5,5-tetramethyl-1,3,2-dioxaborolane [2]

^{1}H NMR, 500 MHz, $CDCl_3$

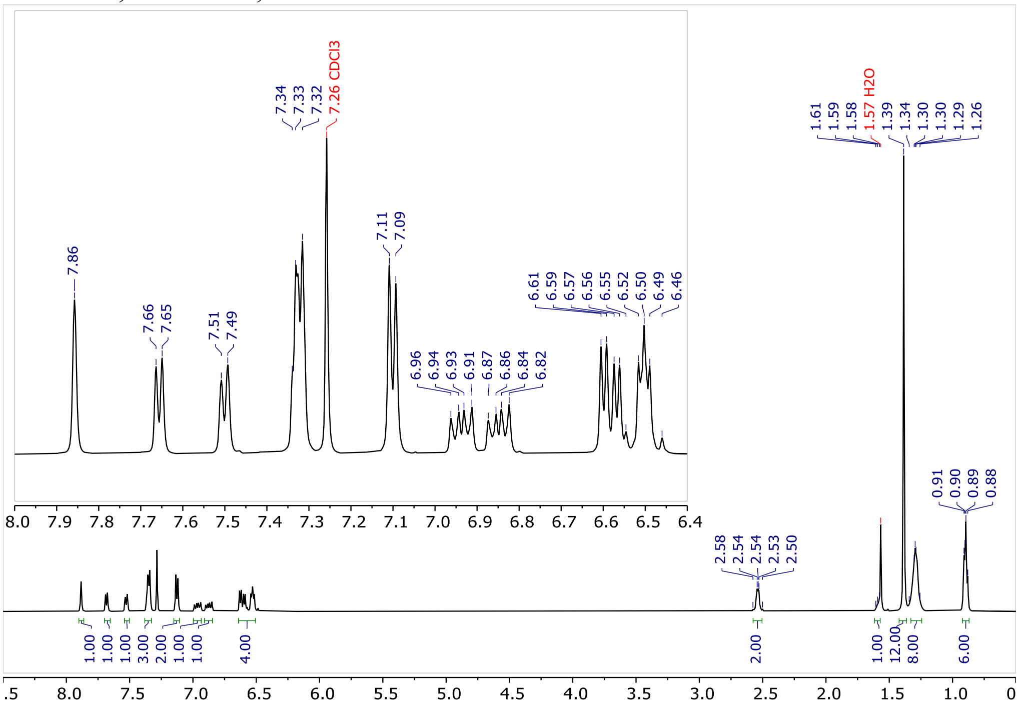

^{13}C NMR, 126 MHz, $CDCl_3$

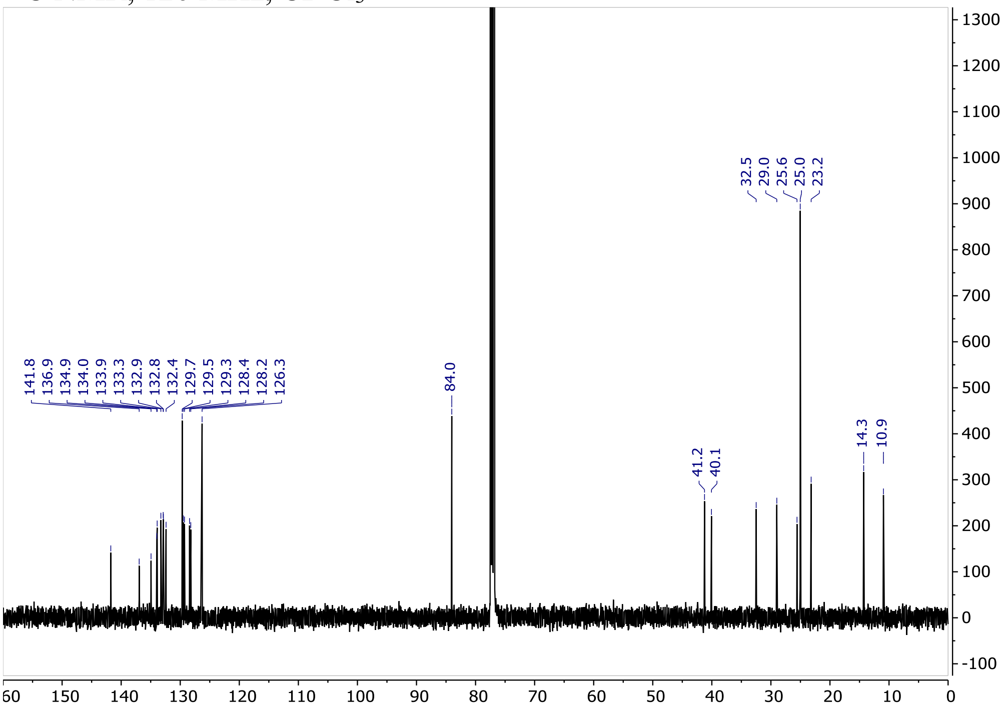

(1E,3E,5E)-1,6-bis(3'-((1E,3E,5E)-6-(4-(2-ethylhexyl)phenyl)hexa-1,3,5-trien-1-yl)-[1,1'-biphenyl]-3-yl)hexa-1,3,5-triene [(DPH)$_3$]

^{1}H NMR, 500 MHz, CDCl$_3$

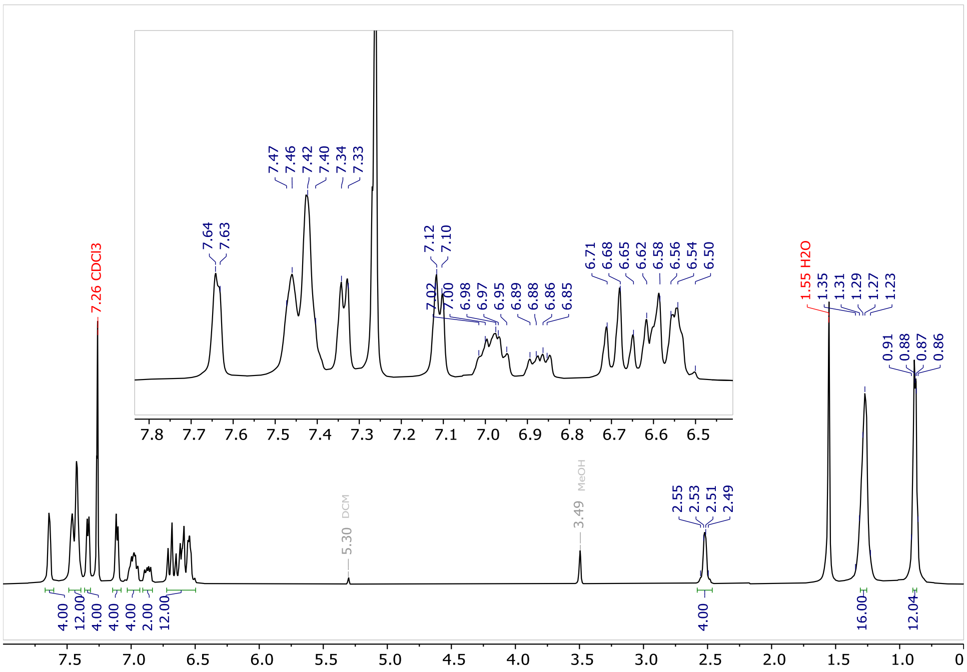

^{13}C NMR, 126 MHz, CDCl$_3$

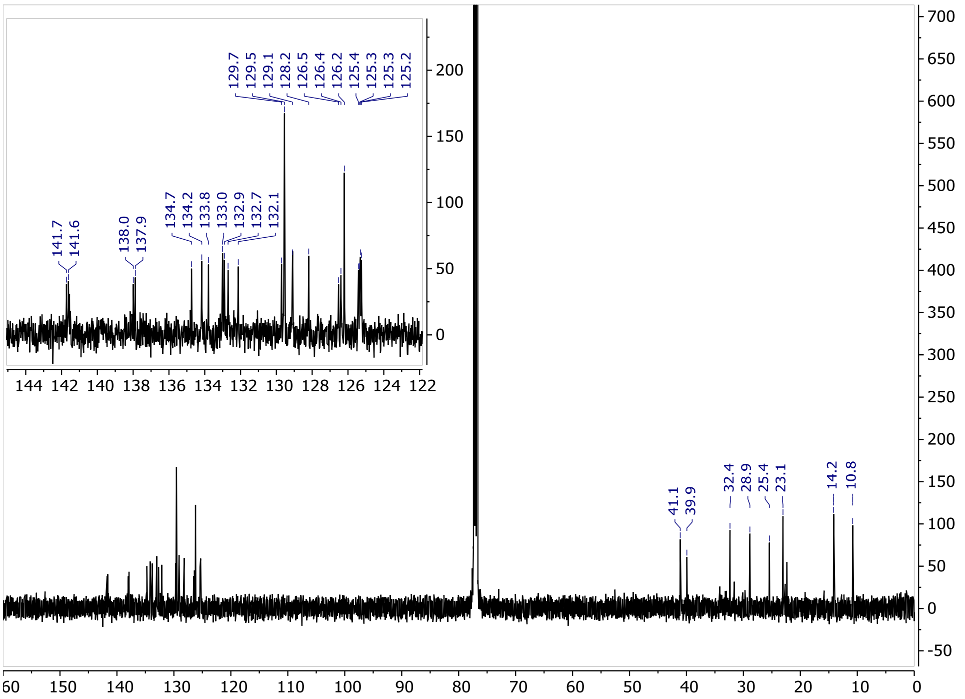

3,3'-((2E,4E,6E)-octa-2,4,6-triene-2,7-diyl)bis(bromobenzene) [3]

^{1}H NMR, 400 MHz, $CDCl_3$

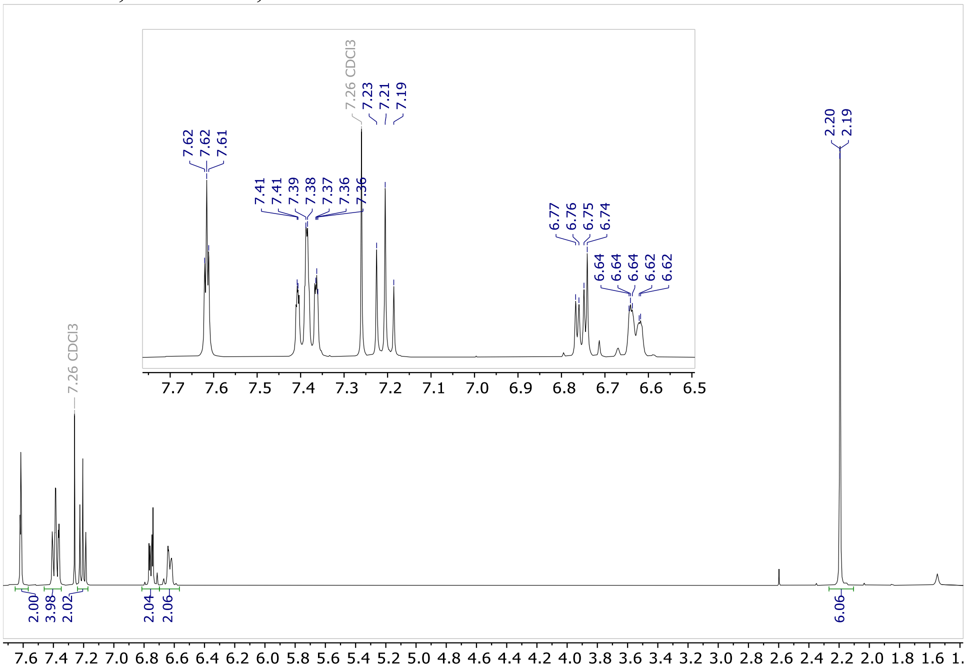

^{13}C NMR, 101 MHz, $CDCl_3$

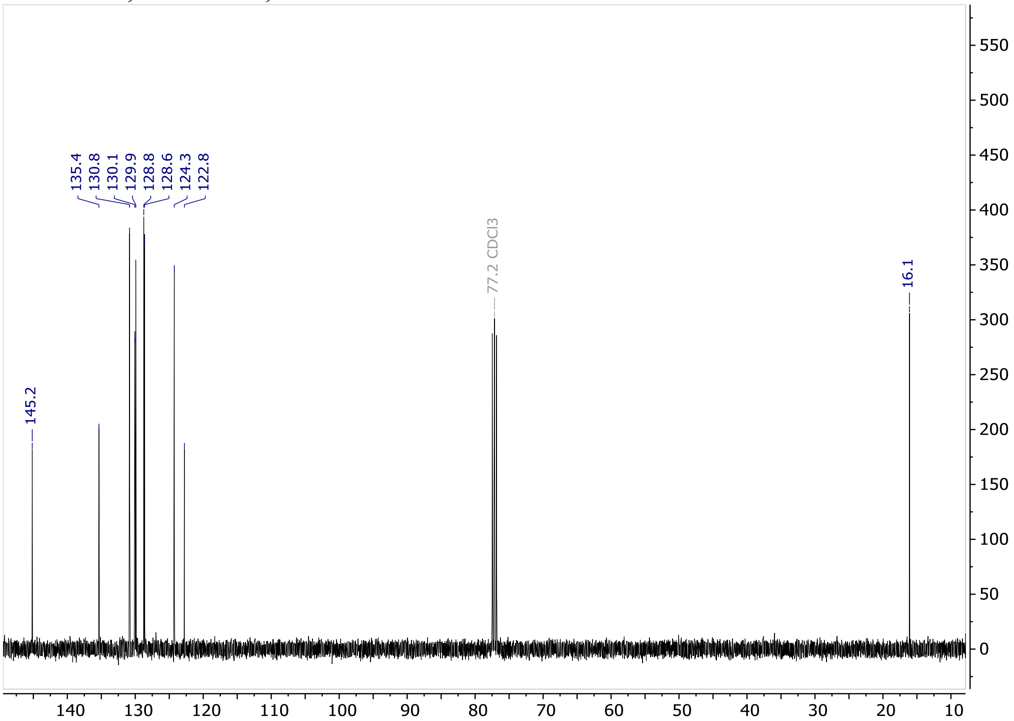

3',3'''-((2E,4E,6E)-octa-2,4,6-triene-2,7-diyl)bis(3-((1E,3E,5E)-6-(4-(2-ethylhexyl)phenyl)hexa-1,3,5-trien-1-yl)-1'',1'''-biphenyl) [Me-(DPH)$_3$]

^{1}H NMR, 500 MHz, $CDCl_3$

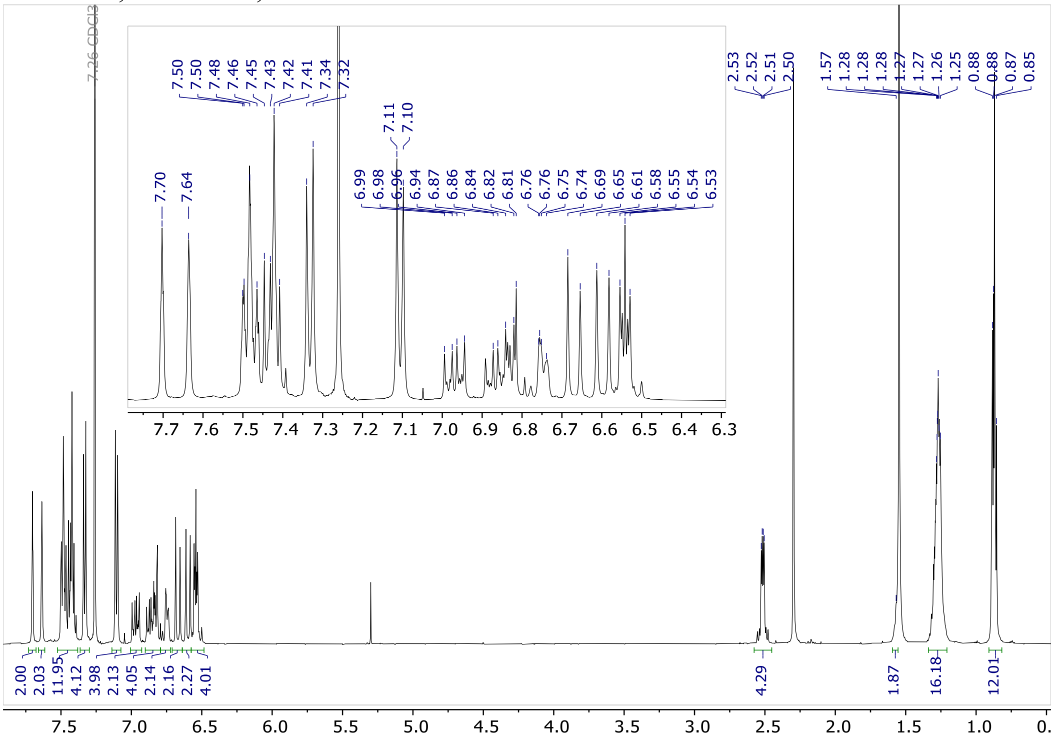

^{13}C NMR, 176 MHz, $CDCl_3$

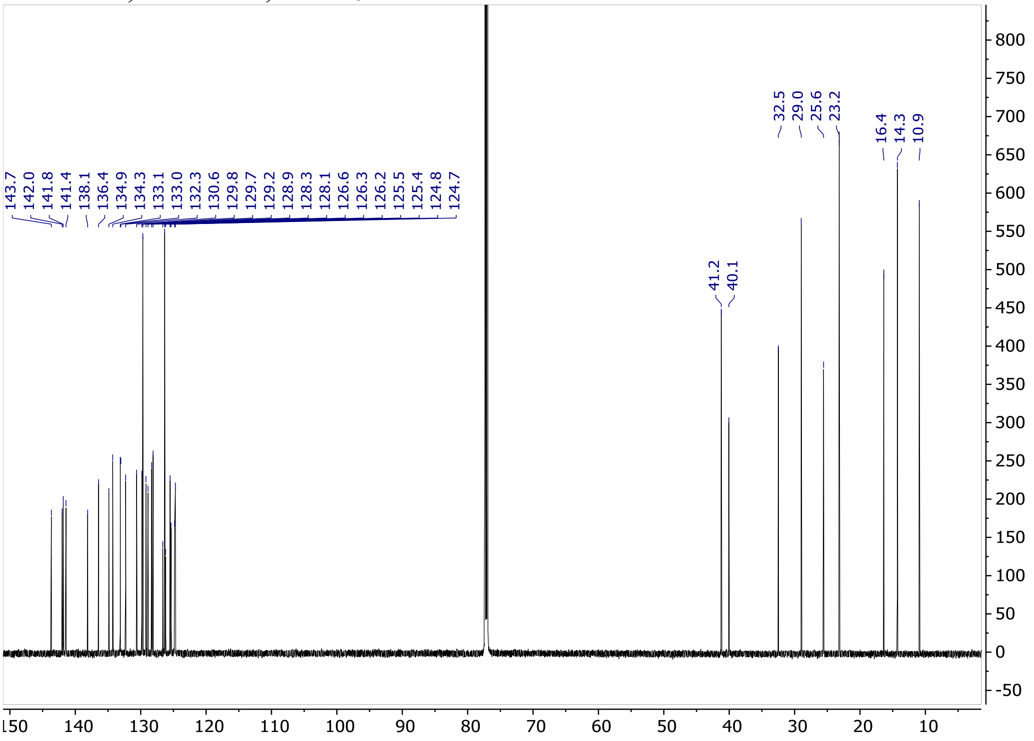

(1E,3E,5E)-1,6-Bis(4-(2-ethylhexyl)phenyl)hexa-1,3,5-triene [DEH-DPH]

^{1}H NMR, 500 MHz, $CDCl_3$

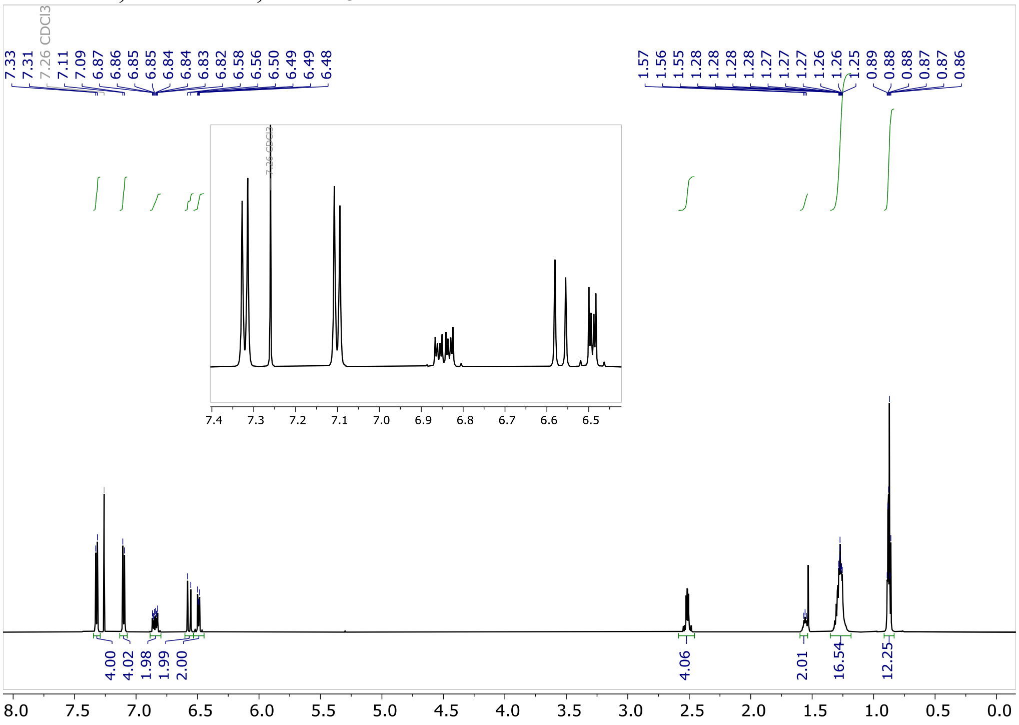

^{13}C NMR, 126 MHz, $CDCl_3$

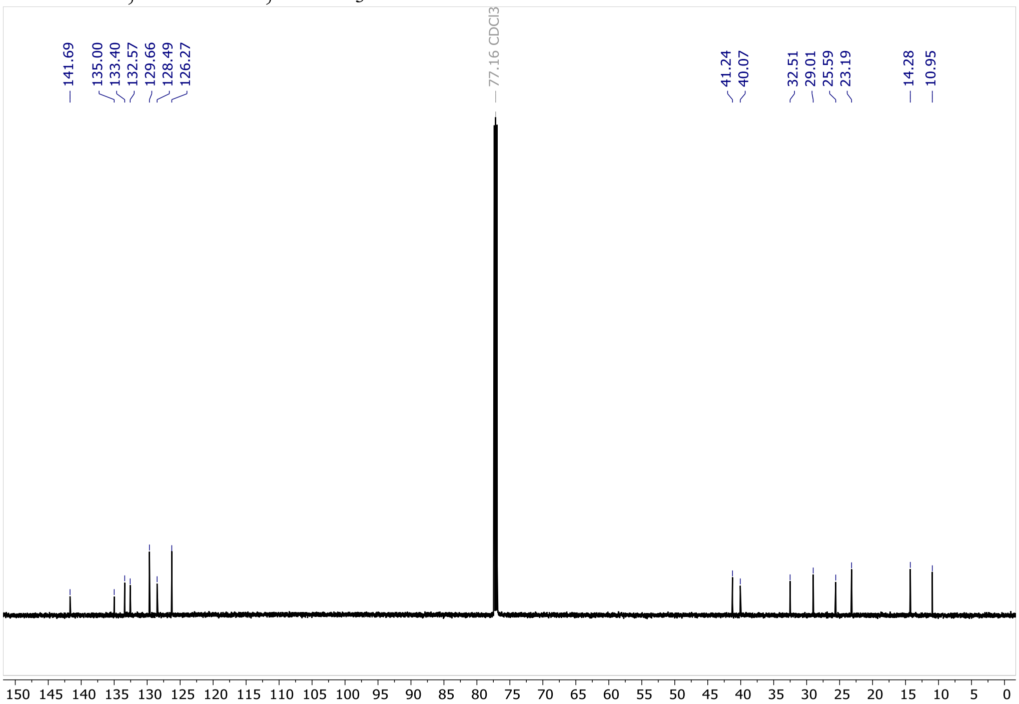

Supplementary References

1 Weiss, L. R. *et al.* Strongly exchange-coupled triplet pairs in an organic semiconductor. *Nature Physics* **13**, 176-181 (2017).

2 Hashimoto, H., Uragami, C., Yukihira, N., Gardiner, A. T. & Cogdell, R. J. Understanding/unravelling carotenoid excited singlet states. *Journal of the Royal Society Interface* **15**, 20180026 (2018).

3 Zheng, C. *et al.* Effect of temperature on Electron-Phonon coupling of carotenoids by Two-Dimensional correlation resonance Raman spectroscopy. *Journal of Molecular Liquids* **365**, 120148 (2022).

4 MacDonald, T. S. *et al.* Anisotropic multiexciton quintet and triplet dynamics in singlet fission via pulsed electron spin resonance. *J. Am. Chem. Soc.* **145**, 15275-15283 (2023).

5 Matsuda, S., Oyama, S. & Kobori, Y. Electron spin polarization generated by transport of singlet and quintet multiexcitons to spin-correlated triplet pairs during singlet fissions. *Chemical Science* **11**, 2934-2942 (2020).

6 Kobori, Y., Fuki, M., Nakamura, S. & Hasobe, T. Geometries and Terahertz Motions Driving Quintet Multiexcitons and Ultimate Triplet–Triplet Dissociations via the Intramolecular Singlet Fissions. *The Journal of Physical Chemistry B* **124**, 9411-9419 (2020).

7 Weber, S. Transient EPR. *eMagRes*, 255-270 (2007).

8 Richert, S., Tait, C. E. & Timmel, C. R. Delocalisation of photoexcited triplet states probed by transient EPR and hyperfine spectroscopy. *Journal of Magnetic Resonance* **280**, 103-116 (2017).

9 Joshi, G. *et al.* Optical readout of singlet fission biexcitons in a heteroacene with photoluminescence detected magnetic resonance. *The Journal of Chemical Physics* **157** (2022).

10 Bayliss, S. L. *et al.* Geminate and nongeminate recombination of triplet excitons formed by singlet fission. *Physical review letters* **112**, 238701 (2014).

11 Yago, T., Ishikawa, K., Katoh, R. & Wakasa, M. Magnetic field effects on triplet pair generated by singlet fission in an organic crystal: Application of radical pair model to triplet pair. *The Journal of Physical Chemistry C* **120**, 27858-27870 (2016).

12 Wang, Z. *et al.* Free-triplet generation with improved efficiency in tetracene oligomers through spatially separated triplet pair states. *Nature Chemistry* **13**, 559-567 (2021).

13 Tayebjee, M. J. *et al.* Quintet multiexciton dynamics in singlet fission. *Nature Physics* **13**, 182-188 (2017).

14 Dillon, R. J., Piland, G. B. & Bardeen, C. J. Different rates of singlet fission in monoclinic versus orthorhombic crystal forms of diphenylhexatriene. *J. Am. Chem. Soc.* **135**, 17278-17281 (2013).

15 Musser, A. J. & Clark, J. Triplet-pair states in organic semiconductors. *Annual review of physical chemistry* **70**, 323-351 (2019).

16 Millington, O. *et al.* The Interplay of Strongly and Weakly Exchange-Coupled Triplet Pairs in Intramolecular Singlet Fission. *J. Am. Chem. Soc.* **146**, 29664-29674 (2024).

17 Hong, Y. *et al.* Steering the multiexciton generation in slip-stacked perylene dye array via exciton coupling. *Nature communications* **13**, 4488 (2022).

18 Fallon, K. J. *et al*. Quantitative Singlet Fission in Solution-Processable Dithienohexatrienes. *J. Am. Chem. Soc.* **144**, 23516-23521 (2022).
19 Millington, O. *et al*. Soluble Diphenylhexatriene Dimers for Intramolecular Singlet Fission with High Triplet Energy. *J. Am. Chem. Soc.* **145**, 2499-2510 (2023).
